# Mimicking Tumor Cell Heterogeneity of Colorectal Cancer in a Patient-derived Organoid-Fibroblast Model

**DOI:** 10.1016/j.jcmgh.2023.02.014

**Published:** 2023-03-02

**Authors:** Velina S. Atanasova, Crhistian de Jesus Cardona, Václav Hejret, Andreas Tiefenbacher, Theresia Mair, Loan Tran, Janette Pfneissl, Kristina Draganić, Carina Binder, Julijan Kabiljo, Janik Clement, Katharina Woeran, Barbara Neudert, Sabrina Wohlhaupter, Astrid Haase, Sandra Domazet, Markus Hengstschläger, Markus Mitterhauser, Leonhard Müllauer, Boris Tichý, Michael Bergmann, Gabriele Schweikert, Markus Hartl, Helmut Dolznig, Gerda Egger

**Affiliations:** 1 Ludwig Boltzmann Institute Applied Diagnostics, Vienna, Austria; 2 Eberhard Karls University of Tübingen Faculty of Mathematics and Natural Sciences, Tübingen, Germany; 3 CEITEC-Central European Institute of Technology, Masaryk University, Brno, Czech Republic; 4 Department of Pathology, Medical University of Vienna, Vienna, Austria; 5 Clinic of General Surgery, Medical University of Vienna, Vienna, Austria; 6 Institute of Medical Genetics, Medical University of Vienna, Vienna, Austria; 7 Comprehensive Cancer Center, Medical University of Vienna, Vienna, Austria; 8 Max Planck Institute for Intelligent Systems, Tübingen, Germany; 9 Division of Computational Biology, School of Life Sciences, University of Dundee, Dundee, United Kingdom; 10 Department of Biochemistry and Cell Biology, Max Perutz Labs, Vienna BioCenter (VBC), University of Vienna, Vienna, Austria; 11 Mass Spectrometry Facility, Max Perutz Labs, Vienna BioCenter, University of Vienna, Vienna, Austria

**Keywords:** Cancer, Co-cultures, Colorectal Cancer, Fibroblasts, Organoids

## Abstract

**Background & Aims:**

Patient-derived organoid cancer models are generated from epithelial tumor cells and reflect tumor characteristics. However, they lack the complexity of the tumor microenvironment, which is a key driver of tumorigenesis and therapy response. Here, we developed a colorectal cancer organoid model that incorporates matched epithelial cells and stromal fibroblasts.

**Methods:**

Primary fibroblasts and tumor cells were isolated from colorectal cancer specimens. Fibroblasts were characterized for their proteome, secretome, and gene expression signatures. Fibroblast/organoid co-cultures were analyzed by immunohistochemistry and compared with their tissue of origin, as well as on gene expression levels compared with standard organoid models. Bioinformatics deconvolution was used to calculate cellular proportions of cell subsets in organoids based on single-cell RNA sequencing data.

**Results:**

Normal primary fibroblasts, isolated from tumor adjacent tissue, and cancer associated fibroblasts retained their molecular characteristics in vitro, including higher motility of cancer associated compared with normal fibroblasts. Importantly, both cancer-associated fibroblasts and normal fibroblasts supported cancer cell proliferation in 3D co-cultures, without the addition of classical niche factors. Organoids grown together with fibroblasts displayed a larger cellular heterogeneity of tumor cells compared with mono-cultures and closely resembled the in vivo tumor morphology. Additionally, we observed a mutual crosstalk between tumor cells and fibroblasts in the co-cultures. This was manifested by considerably deregulated pathways such as cell-cell communication and extracellular matrix remodeling in the organoids. Thrombospondin-1 was identified as a critical factor for fibroblast invasiveness.

**Conclusion:**

We developed a physiological tumor/stroma model, which will be vital as a personalized tumor model to study disease mechanisms and therapy response in colorectal cancer.

## Graphical abstract

**Figure:**
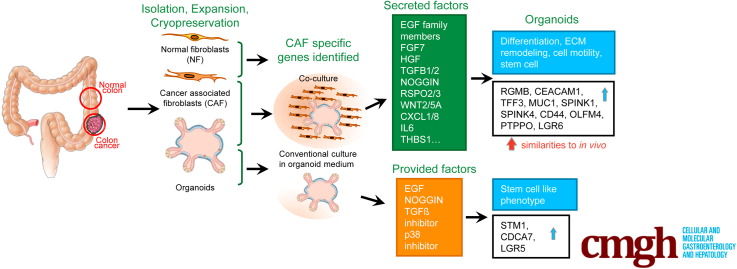

**Summary**

This study presents an advanced tumor organoid model incorporating matched patient-derived tumor cells and fibroblasts. Fibroblasts are capable of supporting the growth of the tumor organoids without the addition of specific niche factors and induce morphological changes in organoids, which render these cultures highly similar to the original tumor tissues.

Colorectal cancer (CRC) remains a main health burden worldwide, being the fourth leading cause of cancer-related deaths.^1^ Although there has been great scientific advance, patient treatment options for advanced CRC remain limited and face poor response prediction. The standard of care therapy has remained unchanged for decades and predominantly relies on chemotherapy, often associated with chemoresistance.^2^

The recent development of advanced tissue culture models, derived from normal as well as tumor tissues, represents an indispensable model reflecting the in vivo tumor characteristics. These so-called patient-derived organoids (PDOs) are promising tools for drug screening and were shown to predict patient response for several tumor entities.^3^ However, CRC organoids generated from metastatic tumors could not predict therapy response of patients for combination therapies with 5-fluorouracil plus oxaliplatin.^4^^,^^5^ This might be due to the specific in vitro culture conditions or the lack of the complex tumor microenvironment (TME) in PDOs. The TME represents the non-malignant part of the tumor and includes infiltrating immune cells, endothelial cells, nerves, and cancer-associated fibroblasts (CAFs). In fact, the TME can have an impact not only on tumor development, but also on therapy response and resistance. Patients with tumors displaying high CAF signatures face lower rates of survival with high statistical prediction.^6^^,^^7^ The consensus molecular subtypes (CMS) of CRC include a mesenchymal subtype CMS4, which is characterized by stromal infiltration, activation of transforming growth factor-beta (TGF-ß) signaling and genes involved in epithelial-to-mesenchymal transition (EMT), angiogenesis, matrix remodeling, and complement-mediated inflammatory system. Furthermore, CMS4 tumors often present with metastases and thus poorer patient survival compared with the other subtypes.^8^

Fibroblasts are the main cell type in the stroma and its main remodelers, as well as a major source of cytokines, growth, and survival factors. Although fibroblasts in healthy tissues become activated only upon damage to assist in tissue repair, CAFs are characterized by a constantly activated ‘wound healing’ phenotype and tumor supportive properties.^9^ Compared with normal fibroblasts (NFs), CAFs exhibit increased proliferation, secretion of extracellular matrix (ECM), and a specific cytokine profile.^10^ CAFs represent a heterogeneous and plastic population of cells that can be derived from diverse cellular resources, including resident fibroblasts, endothelial cells, adipocytes, and others. Recent single-cell RNA sequencing (scRNA-seq) efforts of CRC tumors revealed 2 main CAF populations, including inflammatory (iCAFs) and myofibroblast-like fibroblasts, which are involved in immune-crosstalk and matrix remodeling, respectively.^11^, ^12^, ^13^

Even though specific CAF markers remain elusive, the clinical relevance of CAFs in tumors is indisputable – the abundance of CAFs within a tumor has been associated with poor prognosis in cancer types such as colon^,^^14^^,^^15^ breast,^16^ and pancreas.^17^ Furthermore, there is an extensive cross talk between fibroblasts and other stromal cell types including immune cells, where CAFs exhibit immunosuppressive properties, thus preventing tumor recognition by the immune system.^18^^,^^19^ In fact, early co-culture experiments have demonstrated that irradiated fibroblasts better support tumor cell growth compared with non-irradiated fibroblasts, suggesting that these cells actively affect the tumor and potentially affect response to therapy.^9^^,^^20^, ^21^, ^22^, ^23^ Further, a recent report identified an association of iCAFs with poor prognosis in rectal cancer following irradiation.^24^

Here, we describe the establishment and characterization of an advanced co-culture PDO model, in which CRC-derived epithelial cells and patient-matched fibroblasts can be co-cultured. With this model, we studied the cross talk and dependencies between tumor organoids and NF or CAFs from the same patient. Importantly, this model allows for the cultivation of PDOs without the addition of niche factors such as EGF, R-Spondin1, Noggin, or WNT3a, which we find expressed by fibroblasts, and inhibitors such as SB202190 and A-8301, which are known to affect fibroblast phenotype and proliferation.^25^^,^^26^ We find that PDOs co-cultured with fibroblasts show a heterogeneous cellular composition and closer resemblance to primary tumor tissues based on histomorphology and gene expression profiles inferred from scRNA-seq data using a bioinformatics deconvolution strategy. Overall, this system represents a valuable tool for drug development, precision medicine, therapy-response prediction, and improved understanding of the carcinoma biology in respect to tumor-stroma cross talk.

## Results

### Isolation of Primary Cells and Generation of the Co-culture Model

**Figure 1:**
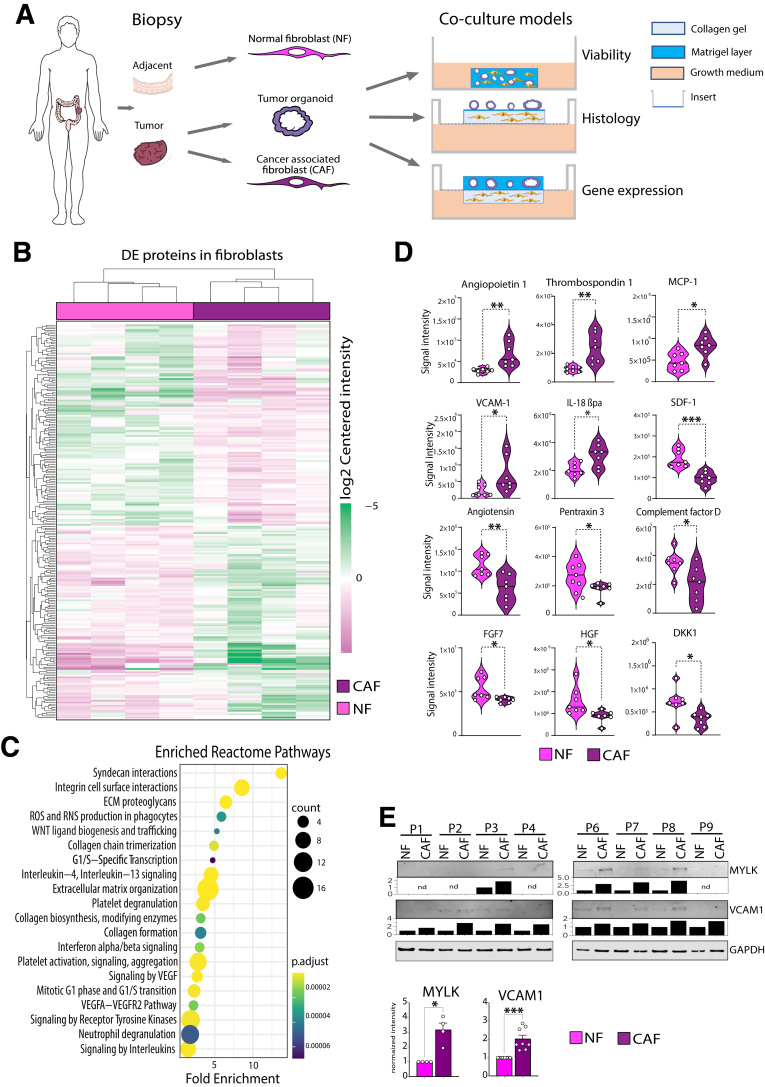
**Molecular and phenotypic differences of NFs and CAFs.** (*A*) NFs and CAFs as well as PDOs are isolated from colon tumors or normal adjacent mucosa. PDOs together with either NFs or CAFs are cultured in different co-culture models containing either Matrigel alone or with collagen I as supportive matrices. Individual setups were used for molecular analyses as indicated (*right*). (*B*) Hierarchical clustering of significantly differentially expressed proteins between 4 individual pairs of NFs and CAFs as analyzed by label free MS/MS (*P* < .05, absolute log2 fold change ≥1) (*C*) Bubble chart describes 20 of the top significant pathways detected by Reactome pathway analysis of differentially expressed proteins. The size of the circles corresponds to the number of proteins changed in the respective pathway. Adjusted *P* values are indicated by different colors according to the color gradient. (*D*) Protein intensity values of significantly differentially secreted proteins between 7 matched NF and CAF pairs as analyzed by human proteome profiler assays (∗*P* < .05, ∗∗*P* < .005, unpaired 2-tailed Student's *t* test). (*E*) Western blot analysis of molecules upregulated in CAFs vs NFs identified by proteomics. Graphs underneath show quantification of the blots by imageJ. Bar graphs indicate fold mean changes compared with the individual NFs (∗*P* < .05, ∗∗∗*P* < .001, unpaired 2-tailed Student's *t* test).

To generate a physiological tumoroid model containing stromal cells, we isolated epithelial tumor cells and fibroblasts from the same patient (Figure 1*A*). Fibroblasts were cultivated in 2D and derived from the tumor and normal adjacent tissues. Organoids were generated in Matrigel following standard protocols.^27^ For co-cultivation, we developed individual setups using matched tumor cells and fibroblasts from individual patients. We generated matching organoids and NFs and CAFs from 6 patients (Table 1). Both tumor cells and fibroblasts were either cultivated together in Matrigel plugs, or tumor cells and fibroblasts were divided into individual layers in air/liquid interface cultures.

**Table 1:** Patient Demographics

Text ID	Gender	Age at resection, *y*	Tumor differentiation	Histological classification	Microsatellite	TNM	Stage at diagnosis	Tumor location	Mutations	APC	TP53	PIK3CA	KRAS	BRAF	FBXW7
P1	M	75	G2	Adenocarcinoma	Stable	pT4b, pN0, L0, V0, Pn0	Iic	Transverse	TP53 (Arg273His), FBXW7 (Arg505Cys)						
P2	F	73	G2	Adenocarcinoma	Stable	pT3, pN0, L0, V0, Pn0	IIa	Coecum	KRAS (Gly12Ala)						
P3	M	64	G2	Adenocarcinoma	Stable	pT2, pN0, L1, V0, Pn0	I	Ascending	KRAS (Ala146Thr), PIK3CA (Glu542Lys)						
P4	F	65	G2	Adenocarcinoma	Stable	pT2, pN0, L0, V0, Pn0	I	Sigma	KRAS (N129S)						
P5	F	81	G2	Adenocarcinoma	Loss of MLH1, PMS2	pT3, pN0, L0, V0	IIa	Coecum	na						
P6	F	78	G3	Mucinous adenocarcinoma	Loss of MLH1, PMS2	pT3, pN0, L1, V0, Pn0	IIa	Ascending	BRAF (Val600Glu), FBXW7 (Asn401Ile/Arg278∗)						
P7	M	57	G2	Adenocarcinoma	Stable	pT4b, pN1b, L1, V1, Pn1	IIIc	Sigma	KRAS (Gly413Asp)						
P8	F	62	G3	Mucinous adenocarcinoma	Stable	pT3, pN1b, L1, V0	IIIb	Sigma	TP53 (His193Arg), KRAS (Gly12Val)						
P9	F	65	G2	Adenocarcinoma	Stable	pT3, pN0, L0, V0, Pn0	IIa	Rectum	TP53 (Arg213∗)						
P10	F	75	G2	Mucinous adenocarcinoma	Stable	pT4a, N0, L1, V0, Pn1	IIb	Sigma	KRAS (Gly12Asp), PIK3CA (Glu542Lys)						
P11	M	56	G2‒G3	Adenocarcinoma	Stable	pT3, pN2b, L1, V1, Pn0	IIIc	Ascending	KRAS (Gly12Asp), TP53 (Arg175His), SMAD4 (Tyr353His)						
P12	F	57	G2‒G3	Adenocarcinoma	Stable	PT3, pN2b, L1, V0, Pn1	IIIc	Sigma	KRAS (Gly12Asp), TP53 (Tyr220Cys)						

Note: Description of clinical and molecular characteristics of patients involved in the study.

F, Female; M, male; MSI, microsatellite instability.

### Characterization of Normal and Tumor Stromal Fibroblasts by Proteomic and Secretome Analysis

As NFs exhibit multiple differences compared with CAFs in vivo, we first investigated whether these variances remained stable in vitro. For this, we collected total protein from early passages of patient-matched NFs and CAFs isolated from adjacent normal tissue and CRC specimens. By label-free tandem mass spectrometry (MS/MS) proteomic analysis of 4 independent patient-matched NF and CAF pairs, a total of 4796 protein groups were detected, of which 235 were significantly differentially expressed (*P* < .05; absolute log2 fold change ≥1.5) (Figure 1*B* and Supplementary Table 1). Reactome pathway analysis revealed that molecules related to ECM organization, collagen biosynthesis, and cell attachment were deregulated in CAFs compared with NFs (Figure 1*C*). Additionally, several immune pathways, such as interleukin or interferon signaling, were significantly affected between the 2 fibroblast groups. Among the top targets, we found proteins related to cell migration and cell contraction, including CEMIP, NES, MYLK, RND3, and SVIL, which were increased 2- to 3-fold in CAFs compared with NFs (Supplementary Table 1).

Secretome analysis of conditioned media of 7 pairs of matched NFs and CAFs using cytokine arrays identified 12 molecules that were significantly deregulated between CAFs and NFs (Figure 1*D*). Among the top differentially secreted molecules were MCP-1 (CCL2) and thrombospondin 1 (THBS1) (both increased in CAFs), as well as DKK1 and PTX3 (both increased in NFs). MCP-1 is a promoter of lung metastasis in breast cancer by promoting angiogenesis,^28^ and we recently showed its important function for the recruitment of monocytes to the TME.^29^ THBS1 is a TGF-β activator and has been found overexpressed in the tumor stroma.^30^ DKK1 is an antagonist of Wnt signaling, which is downregulated at the onset of adeno-carcinoma formation.^31^ Finally, the exact role of PTX3 in cancer has not been fully elucidated; however, it was suggested as an oncosuppressor in mice and humans via regulation of complement-dependent tumor-promoting inflammation.^32^ In addition, we validated the differential expression of the myofibroblast marker MYLK and the vascular cell adhesion molecule VCAM1 using Western blot analysis (Figure 1*E*).

Together, these data show that NFs and CAFs retain their functional proteome and secretome in vitro, which reflects tumor-associated pathways in CAFs.

### Collagen Motility Assay Reveals Phenotypic Differences Between CAFs and NFs

**Figure 2:**
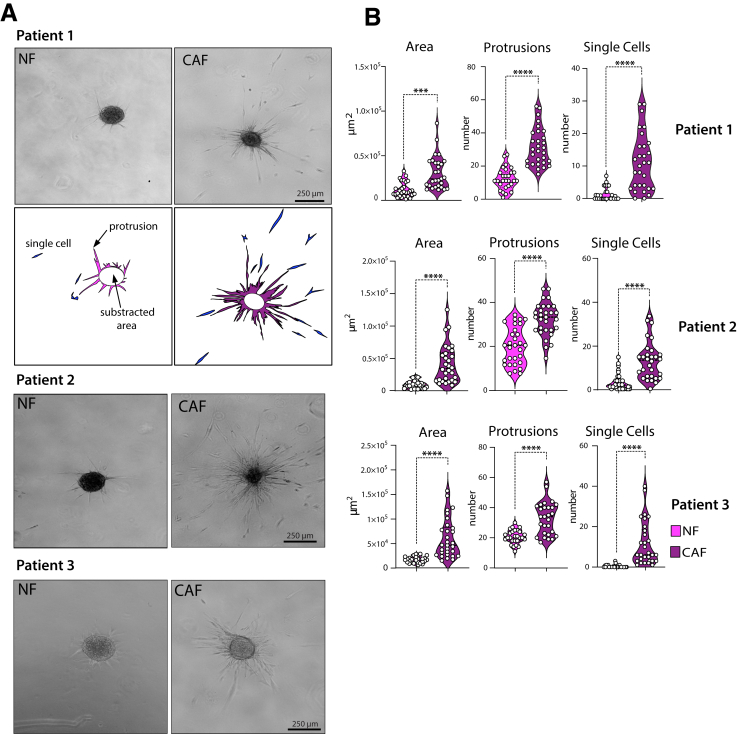
**Fibroblasts morphology in a collagen gel motility assay**. (*A*) Microscopic pictures of matched NFs and CAFs isolated from 3 individual patients embedded as spheroids into collagen I for 24 hours. Illustration in lower panel of patient 1 shows method of quantification of the sprouting area, number of protrusions, and single cells in CAFs vs NFs. (*B*) Violin plots show measurements of the sprouting area (total area minus spheroid core area), number of protrusions, and number of single cells detached from the spheroids in the 3 individual patients. Experiments were performed in triplicates, and >10 individual spheroids were analyzed for each experiment (n = 30) (*∗P* < .05, *∗∗P* < .005, ∗∗∗*P* < .001, 2-tailed unpaired Student's *t* test).

Based on the proteome and secretome data, we evaluated whether these molecular changes translate into phenotypic differences between NFs and CAFs. Indeed, 3D collagen gel motility assays of fibroblast spheroids^33^ revealed significantly higher spread areas, numbers of protrusions, and numbers of single cells detached from the spheroid structures in CAFs compared with NFs (Figure 2*A–B*) in 3 matched early passage NF and CAF pairs. These findings suggest that the alterations found in the proteomic analyses indeed led to a phenotypic difference manifested by increased motility of CAFs in collagen gels.

### Normal and Cancer-associated Fibroblasts Support Organoid Growth in Co-culture

**Figure 3:**
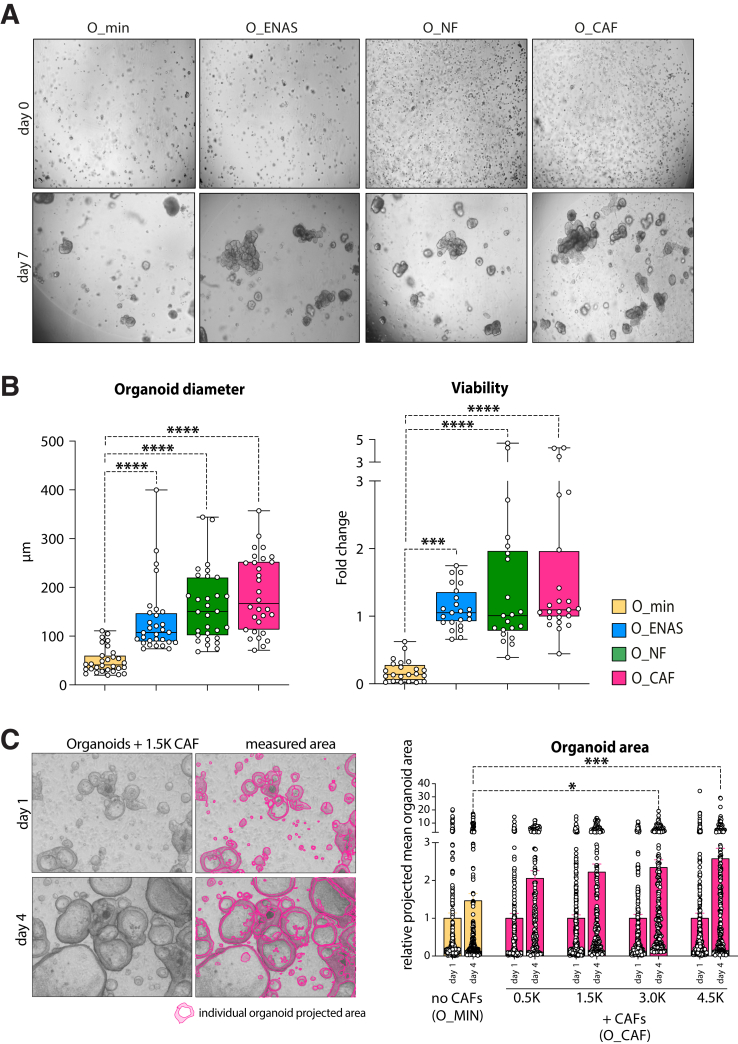
**Organoid growth and morphology in co-cultures with fibroblasts.** (*A*) Representative microscopic pictures of organoids grown in Matrigel for 7 days in minimal medium (O__min_), conventional organoid medium (O_ENAS), or together with NFs (O_NF) or CAFs (O_CAF) starting from single cell suspensions. (*B*) On day 7, organoid diameters were measured using Fiji imaging software, and viability was analyzed using CellTiter-Glo 3D assay (n = 3 patients) in 7 to 8 technical replicates. (*C*) Dependence of organoid growth related to fibroblast numbers. Images on the left show growth of organoids over time and how the organoid growth area was measured. Graph on the right represents comparison of mean organoid area (from 4 frames/condition) in presence of different numbers or absence of fibroblasts (*∗∗∗P* < .001, ∗∗∗∗*P* < .0001; 2-tailed unpaired Student's *t* test).

Following NF and CAF characterization, we sought to investigate the crosstalk between tumor cells and stromal fibroblasts. For this, we established a model where fibroblasts and PDOs derived from the same patient were co-cultured in Matrigel drops in minimal medium (1% fetal bovine serum [FBS]). We omitted the addition of any niche factors or inhibitors that are usually added to conventional organoid growth medium. Importantly, these factors were shown to alter fibroblast signaling and phenotype.^34^^,^^35^ Particularly, inhibitors of TGF-β signaling (A-8301) or cell cycle (SB202190)^36^ would potentially mask intrinsic signals in the primary fibroblasts, thus influencing cell-cell crosstalk between stromal cells and tumor organoids. Of note, control organoids, which carried mutations in the *APC* gene, were grown in medium without supplementation of Wnt3a and R-spondin1 to enrich for tumor cells, as previously described.^37^ Indeed, the viability and size of organoids co-cultured in the minimal medium with matched fibroblasts (O_CAF, O_NF) were comparable to these of organoids cultivated in regular organoid medium (O_ENAS) (Figure 3*A–B*) and showed dose-dependent growth in response to the number of co-cultivated fibroblasts (Figure 3*C*). Long-term organoid growth was not supported in minimal medium alone (O_min). This indicated that factors derived from NFs or CAFs were equally potent to support organoid growth and survival as compared to conventionally used organoid medium.

### Organoid-fibroblast Co-cultures Closely Represent Patient Histology

**Figure 4:**
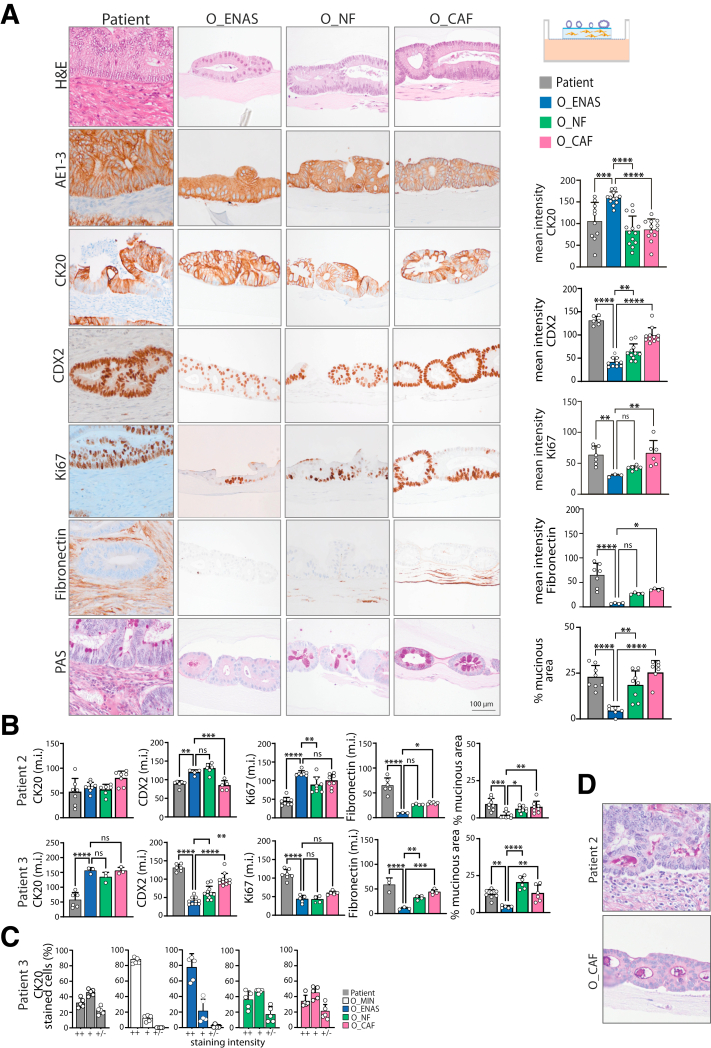
**Organoid morphology in co-cultures with fibroblasts.** (*A*) Illustration on the top right displays the experimental setup. Comparison of the histo-morphology of a representative set of matched patient material and organoids grown either alone in ENAS medium or in co-culture with NFs or CAFs in minimal medium. After cultivation for 7 days, the matrices were formalin-fixed and paraffin-embedded and sectioned into 2-μm thick slices. Consecutive slices were stained with hematoxylin and eosin (H&E), Periodic acid-Schiff (PAS) stain or via immunohistochemistry with indicated antibodies (AE1-3, pan-keratin; CK20, cytokeratin 20; CDX2, differentiation marker; Ki67, proliferation marker; Fibronectin, fibroblast marker). Graphs on the right represent quantification of the images using Fiji software. A minimum of 3 images were quantified for each graph. Values presented are means ± standard deviation, ∗*P* < .05, ∗∗*P* < .005, ∗∗∗*P* < .001, ∗∗∗∗*P* < .0001; ordinary 1-way ANOVA. (*B*) Quantification of immunohistochemichal stainings as in (*A*) for 2 additional matched patient/organoid sets. (*C*) Quantification of intensity of CK20 staining for one patient shown in (*B*), ++ high, + medium and ± low intensity. Data are mean values ± standard deviation. (*D*) PAS stain of patient 2 tumor tissue and the matched organoid/CAF co-culture, indicating mucus production in both the tumor tissue and organoid culture without the presence of goblet-like cells.

Next, we evaluated whether the fibroblast/organoid co-culture model recapitulated the histological properties of the primary patient tumor. For this purpose, we embedded fibroblasts in a collagen I gel, which was coated with Matrigel and plated organoids on top. This setup allows for direct cell-cell interaction and mirrors closely the in vivo tissue architecture of the colon. We used specific staining methods and immunohistochemistry such as hematoxylin and eosin and periodic acid–Schiff staining, as well as immunohistochemical stainings with antibodies against CK20, CDX2, Ki67, and pan CK (Figure 4*A–B*). We compared the histomorphology of the original resected tumor with NF and CAF co- and mono-cultured organoids. The co-cultures of the organoids with NFs and CAFs showed comparable morphology, which was highly similar to the patient material. Organoids grown without fibroblasts in ENAS medium appeared slightly different, displaying a more homogenous expression of certain markers, including CK20 and Ki67. As expected, fibronectin, a marker of connective tissue cells and CAFs, was clearly present in the fibroblast layer of the co-cultures and in the stroma of the primary tumor but not in the ENAS organoid cultures. Particularly, the heterogeneity in expression of the intestinal cell differentiation marker CK20 in the patient samples was very closely recapitulated in the fibroblast co-cultures. Thus, the epithelial organoid structures in the CAF and NF co-cultures displayed the same distribution of undifferentiated, intermediate, and highly differentiated cells as in the patient sample. In contrast, the organoids in ENAS medium displayed less similarity to the primary tumor and showed a more even CK20 expression based on quantification of positively stained cells (Figure 4*C*). Strikingly, periodic acid–Schiff staining revealed that the percentage of mucus producing cells in the patient tumors was similar to the one in the in vitro co-cultures. However, mucus production was less pronounced in the organoids grown in full organoid medium without fibroblasts. In one of the tumors, mucus was evident without the presence of clearly distinguishable mucus-producing goblet-like cells, which was also reflected in the co-cultures (Figure 4*D*). These data suggest that organoids co-cultured with NFs or CAFs manifest a significant similarity to the original in vivo tumor, as indicated by the presence of various cell types.

### RNA Sequencing Reveals Deregulated Genes in Organoids Grown With Fibroblasts or ENAS Medium

**Figure 5:**
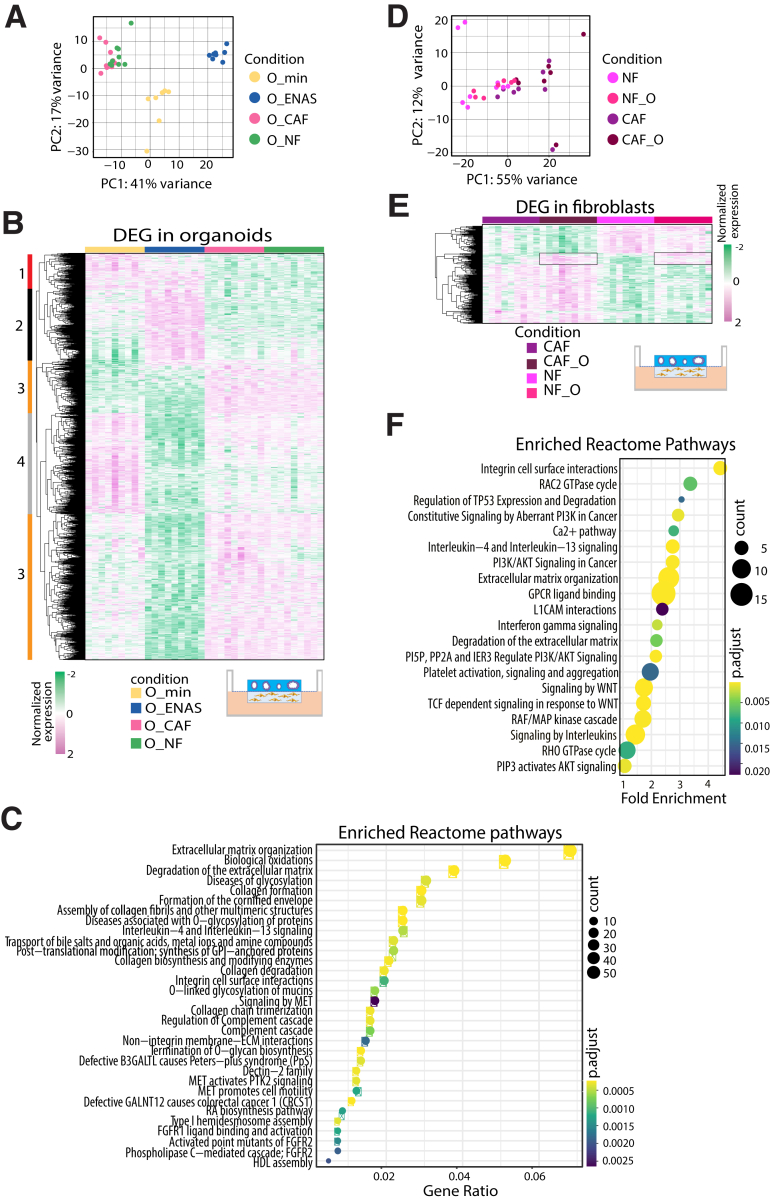
**Gene expression analysis of tumor organoids and fibroblasts.** (*A*) PCA plot of organoid samples grown in minimal medium (O__min_), conventional organoid medium (O_ENAS), or together with NFs (O_NF) or CAFs (O_CAF) plotted in 2 dimensions using their projections onto the first 2 principal components based on RNA-seq data. The different conditions are color coded. (*B*) Hierarchical clustering of significantly differentially expressed genes in organoids grown in different conditions as in (*A*) as identified from RNA-seq analyses. Numbers and colors on the left of the heatmap indicate 4 different groups of genes that were upregulated in O__min_ (1, *red*), upregulated in O_ENAS (2, *black*), upregulated in the co-cultures O_NF and O_CAF (3, *orange*), or downregulated in O_ENAS (4, *grey*). The illustration below the heatmap shows the experimental setup. (*C*) Reactome pathway analysis shows the top significantly altered pathways between the individual growth conditions of organoids. Size of circles defines the number of genes affected in the respective pathway and the color indicates the significance (adjusted *P* value) according to the color gradient shown. (*D*) PCA plot of fibroblasts including NFs and CAFs grown either alone (NF, CAF) or in co-culture with tumor organoids (NF_O, CAF_O) (indicated by colors) plotted in 2 dimensions using their projections onto the first 2 principal components based on RNA-seq data. (*E*) Hierarchical clustering of significantly differentially expressed genes of NFs and CAFs grown in different conditions as in (*D*). Rectangles on the heatmap mark genes that were specifically upregulated in the co-culture conditions in NFs and CAFs compared with the mono-cultures. (*F*) Bubble chart lists the top significantly changed pathways using Reactome pathway analysis in fibroblasts based on the different culturing conditions. The number of genes involved in the respective pathway is shown by the size of the circles and the significance by the color according to the color gradient. The RNA-seq analyses were performed in triplicates for 3 individual patients (P1‒3) (9 replicates in total) (*P* adj. < .05).

To dissect the crosstalk between stromal fibroblasts and epithelial tumor cells, we co-cultured organoids with patient matched fibroblasts for 4 days in a 3D setting and compared it with the single cultures of organoids or fibroblasts. In a modular setup, organoids were cultured in Matrigel domes and fibroblasts in collagen I gels; however, the gels were positioned on the opposite sides of a transwell membrane insert, allowing separate cultivation in the presence of soluble factor exchange and direct cell-cell interaction via the 0.45 μm diameter membrane pores as previously shown.^38^ After separation, the individual gels were lysed, and mRNA was prepared to perform bulk RNA sequencing (RNA-seq). We compared organoids grown in ENAS condition with organoids cultivated in combination with NFs or CAFs and organoids cultivated in minimal medium (O__min_), which was also used in the co-cultures. The high purity of the fibroblast and tumor cell populations was confirmed by selective expression of cell type-specific genes including *CDH1*, *EPCAM*, and *KRT8* for tumor cells and *VIM*, *DCN*, *THY1*, and *COL3A1* for fibroblasts (Supplementary Table 2). Interestingly, principal component analysis (PCA) of the transcriptomes of organoids grown in the different conditions showed a clear separation of organoids grown in ENAS or minimal medium from the organoid/fibroblast co-cultures (Figure 5*A*). Among the organoid fraction from the different conditions, we identified a total of 1742 significantly deregulated genes from pairwise comparison of the 4 different conditions (Supplementary Table 3). Supervised clustering of these deregulated genes revealed separate gene expression groups (Figure 5*B*). The first group included genes upregulated in the O__min_ and ENAS conditions but downregulated in the co-cultures, which were involved in MAPK1/MAPK3 signaling and PI3K/AKT signaling, as well as signaling by interleukins. The second group of genes was specifically upregulated in the ENAS condition and contained genes involved in EGFR and FGFR signaling as well as cell-cycle regulation genes. In the third group of genes, we observed genes upregulated in both co-culture conditions and downregulated in the ENAS and O__min_ conditions. This large group contained genes involved in ECM organization, collagen synthesis, and modifying enzymes, as well as genes involved in innate immune system pathways. Finally, genes upregulated solely in the O__min_ condition, involved metabolism and differentiation associated genes. Reactome pathway analysis based on all 1742 significantly deregulated genes identified top altered pathways related to collagen biosynthesis and ECM organization (Figure 5*C*). Interestingly, several of the identified pathways were related to MET and FGFR signaling and cell motility. Thus, co-cultivation of fibroblasts with tumor organoids is strongly affecting ECM remodeling and cell-cell communication pathways in the tumor cells.

### Co-culturing of NFs and CAFs With Tumor Organoids Induces Expression of Specific Genes

Next, we focused on the fibroblast expression profiles that were either cultured separately or in co-culture with the organoids. Using PCA analysis, we found an overall separation between NFs and CAFs, with no clear segregation of NFs and CAFs cultured with organoids (Figure 5*D*). Similarly, hierarchical clustering revealed that the majority of differentially expressed genes allowed for the separation of NFs vs CAFs independent of culture condition (mono- or co-culture) (Figure 5*E*). We identified 229 significantly differentially expressed genes by pairwise comparison of the 4 conditions (Supplementary Table 4). Within the top differentially expressed genes between NFs and CAFs, we found *WNT2*, *WNT5a*, *VCAM1*, and *WISP1* upregulated in CAFs. These genes were previously identified as bona fide CAF markers in vivo and in vitro.^33^^,^^39^, ^40^, ^41^ In NFs, we found *RSPO3*, *TMEM35A*, and *C7* enriched. *RSPO3* can potentiate the Wnt/β-catenin pathway independent of LGR binding.^42^ *TMEM35* codes for a transmembrane protein 35 that is preserved across species^43^; however, its exact function and particularly its role in cancer is poorly understood. The gene *C7* encodes for complement factor 7 and belongs to a family of proteins involved in immune surveillance and homeostasis. Additionally, C7 was identified as a tumor suppressor in ovarian cancer and non-small-cell lung cancer.^44^ Reactome pathway analysis revealed that a majority of molecules deregulated between NFs and CAFs belong to ECM rearrangement, cell motility, and WNT signaling, confirming our proteomic data (Figure 5*F*). Additionally, cancer pathways such as PI3K/AKT signaling and immune relevant pathways such as interleukin signaling were significantly enriched.

**Figure 6:**
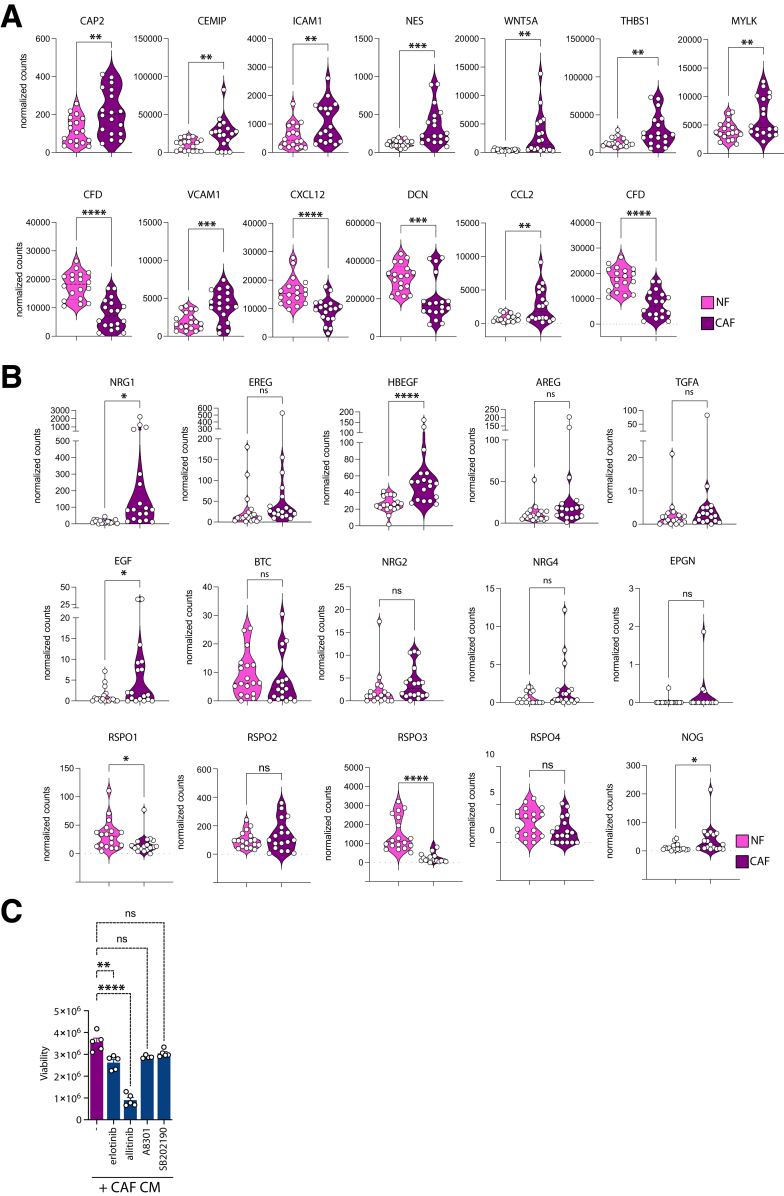
**Validation of gene expression signatures and factor dependencies in fibroblasts.** (*A*) RNA expression levels of selected target proteins significantly deregulated in proteome analysis between NFs and CAFs represented as violin plots. Values plotted represent normalized mRNA counts derived from RNA-seq expression analysis of matched NFs and CAFs derived from 3 individual patients. Data of NFs grown alone or together with organoids and CAFs grown alone or together with organoids from the 3 patients were combined and repeated in 3 technical replicates. (*B*) RNA expression levels of molecules related to factors found in the ENAS medium. Values plotted represent normalized counts as in (*A*). (*∗P* < .05, *∗∗P* < .005, ∗∗∗*P* < .001, ∗∗∗∗*P* < .0001; 2-tailed unpaired Student's *t* test). (*C*) Organoid viability measured by CellTiter-Glo 3D. Organoids were treated with the EGF-receptor inhibitors erlotinib or allitinib, with the TGF-ß inhibitor A-8301 or with the p38 MAP kinase inhibitor SB202190 for 1 week.

Interestingly, we identified a group of 20 genes, which were specifically induced in both NFs and CAFs upon co-culture with organoids in all 3 patients analyzed (boxed in Figure 5*E* and Supplementary Table 4). These genes, including *IL6, ICAM-1*, and *CXCL14*, are associated with immune cell signaling, tumor progression, and metastasis, indicating that tumor cells induce a highly specific gene expression signature to enforce their tumorigenic properties in the TME. In addition, we were able to validate differential gene expression of proteins and secreted factors, which we identified based on proteome and secretome analyses (Figure 6*A*). Interestingly, fibroblasts also highly expressed different growth factors and signaling molecules, including EGF family members, R-Spondins, and Noggin, which are added to classical organoid media (Figure 6*B*). Treatment of organoids grown in CAF conditioned medium (CAF-CM) with the EGFR1-specific inhibitor erlotinib or the pan-EGFR inhibitor allitinib revealed significantly reduced growth upon treatment. In contrast, treatment with the TGF-ßR inhibitor A-8301 or the p38 MAP kinase inhibitor SB202190 had no significant effects on organoid proliferation (Figure 6*C*). Together, these data suggest that fibroblasts support the growth and proliferation of organoids through secretion of a multitude of different growth factors and cytokines.

### Organoids Co-cultured With Fibroblasts Manifest Comparable Gene Expression to Primary Tumors

**Figure 7:**
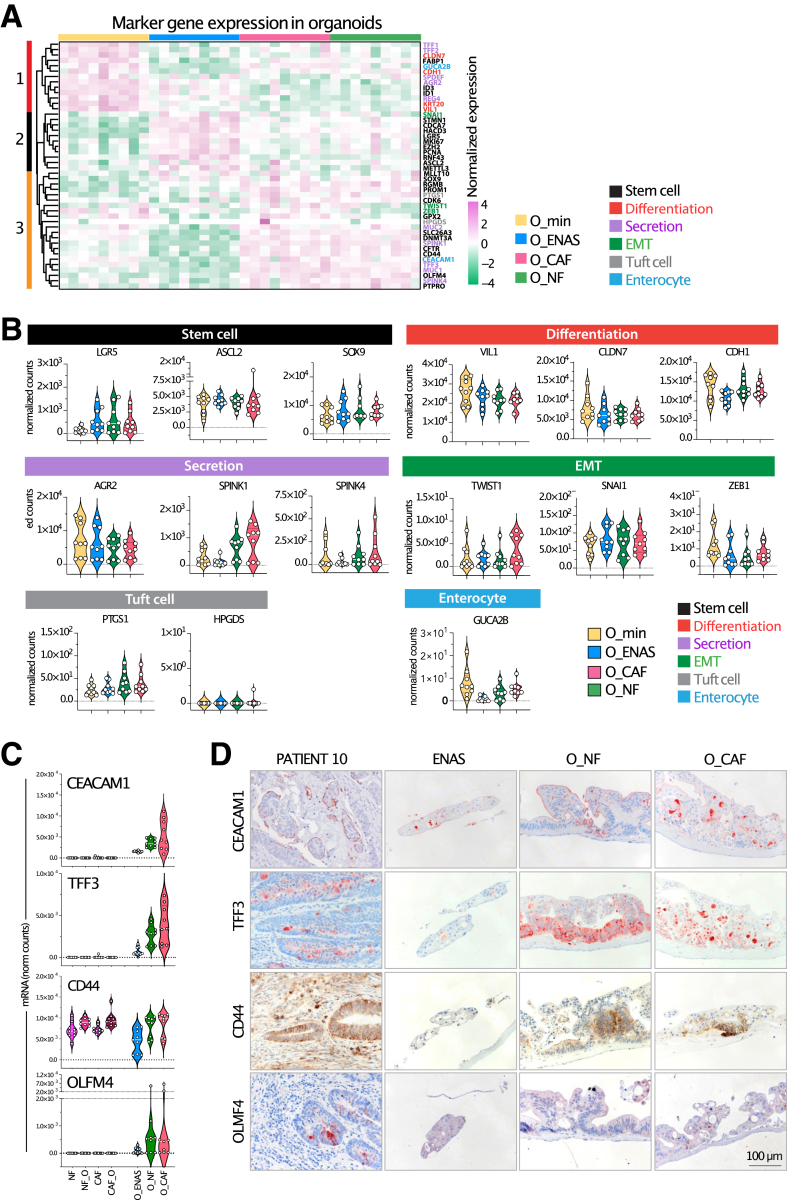
**Expression of epithelial marker genes in organoids.** (*A*) Hierarchical clustering of normalized gene expression levels of individual marker genes derived from published scRNA-seq datasets of primary tumors^12^^,^^13^ in organoids grown in minimal medium (O__min_), conventional organoid medium (O_ENAS), or together with NFs (O_NF) or CAFs (O_CAF). Colored rectangles and numbers on the left indicate genes upregulated in O__min_ (1, *red*), O_ENAS (2, *black*), or in the co-cultures with NF and CAF (3, *orange*). (*B*) mRNA expression levels of deregulated genes, representing different biological features, identified by bulk RNA-seq of organoids in the different culture conditions as in (*A*). (*C‒D*) Validation of selected RNA-seq deregulated target genes by immunohistochemical staining. mRNA expression levels of respective genes are shown in (*C*). Stainings were performed on patient tissues or on the matched cultured organoids grown alone in ENAS medium or with NF (O_NF) or CAF (O_CAF) as co-culture. Antibodies against CEACAM1, TFF3, CD44, olfactomedin 4 (OLMF4) were used (*D*).

To validate the relevance of the organoid-fibroblast model in primary tumors, we compared the gene expression signatures of our dataset to 2 published scRNA-seq datasets of human colorectal tumors.^12^^*,*^^13^ From these datasets, we inferred fibroblast and epithelial marker genes and analyzed their expression in the bulk RNA-seq datasets from mono- and co-cultured organoids and fibroblasts. Among the selected marker genes, we identified 3 different gene groups: (1) genes elevated in O__min_, (2) genes overexpressed in ENAS, and (3) genes upregulated in the co-cultures compared to the mono-cultures (Figure 7*A* and Supplementary Table 5). As expected from the previous analyses, organoids grown in minimal medium (O__min_) showed a strong upregulation of differentiation specific genes such as *CLDN7*, *CDH1*, *KRT20*, or *VIL1.* Also, genes specific for secretion such as *REG4*, *TFF1*, and *TFF2* were upregulated in this condition. Genes specifically upregulated in the ENAS condition included the stem cell-specific genes *STMN1*, *CDCA7*, and *LGR5*, in agreement with the stem cell-supporting properties of the organoid expansion medium. In organoids co-cultured with fibroblasts, we identified stem cell, goblet cell, tuft cell, and enterocyte-specific markers, including *RGMB*, *CEACAM1*, *TFF3*, *MUC1*, *SPINK1*, *CD44*, *OLFM4*, *SPINK4*, and *PTPRO* (Figure 7*B*). To validate the expression of the encoded proteins of selected marker genes (Figure 7*C*), we performed immunohistochemistry in patient specimens and their corresponding organoids, grown in ENAS medium or in co-culture with fibroblasts (Figure 7*D*). Overall, there was closer resemblance between the patient samples and the co-cultures. The expression of trefoil factor 3 (TFF3) and CEACAM1 in the co-cultures was comparable to the patient samples and overall higher compared with the organoids alone. In the primary tumors, CD44 was equally detected in the epithelia and in the stroma, similar to the organoid-fibroblast cultures. OLFM4 showed variable expression both in the patients and the corresponding co-cultures. Finally, MUC1 staining was generally weaker in the co-cultured organoids compared with the patient tissue, but not detectable in the organoid monocultures.

Interestingly, there was no significant difference between genes expressed in organoids grown with NFs or CAFs except for *GUCA2B* and *FABP1,* which were slightly upregulated in the CAF condition. Overall, these data confirm the histomorphological analyses, indicating a shift from a stem cell-like to a more heterogeneous pattern of cells in organoid co-cultures, which might be more closely related to the in vivo tumor situation.

### Fibroblasts in Mono- and Co-cultures Express Similar Gene Signatures to Primary Tumors

**Figure 8:**
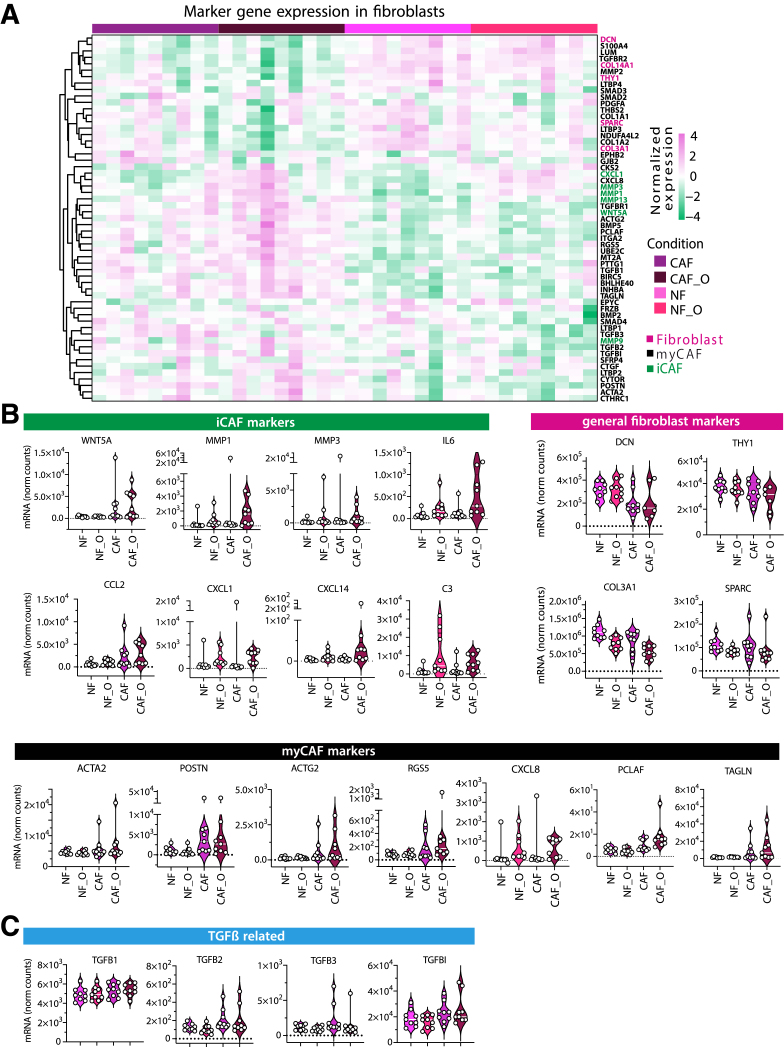
**Expression of fibroblast marker genes.** (*A*) Hierarchical clustering of general fibroblast, myofibroblast (myCAF), and inflammatory fibroblast (iCAF) markers (described in published scRNA-seq datasets of patient tumors^12^^,^^13^^,^^24^ in cultured NFs and CAFs grown alone (NF, CAF) or in co-culture with organoids (NF_O, CAF_O). (*B*) mRNA expression levels of differentially regulated fibroblast marker genes identified by bulk RNA-sequencing of fibroblasts. (*C*) Gene expression levels of TGF-ß-related genes extracted from bulk RNA-seq data as in (*B*).

For the fibroblast markers, we derived markers for general fibroblasts, inflammatory fibroblasts, and myofibroblasts from published sc-RNA-seq data^12^^,^^13^^,^^24^ and compared them with the bulk RNA-seq data from NFs and CAFs grown in the different conditions (Figure 8*A* and Supplementary Table 6). As described above, major differences in fibroblast gene expression could be observed between NF and CAF, independent of the culture condition (mono vs co-culture). However, we also identified several genes, including *WNT5a*, *PCLAF*, *TAGLN, TGFB1*, *CXCL1, CXCL8*, or *MMPs* that were significantly upregulated in CAFs upon co-culture with the organoids (Figure 8*B*). In fact, WNT5a has been previously reported as highly expressed in CAFs^45^ and has a role in cancer progression. iCAF marker genes, including *CXCL1*, *CXCL8*, *CXCL14*, *IL6*, *MMP1*, and *MMP3*, were present at higher levels in the co-culture conditions (both NF and CAF), representing genes inducing proinflammatory and ECM-degrading phenotypes in fibroblasts. These genes were defined as iCAF markers in scRNA-seq analysis.^24^ Interestingly, some of the genes involved in collagen organization, including *DCN*, which has been associated with tumor suppressive activities, or *COL3A1* were downregulated upon co-culture with organoids in both NFs and CAFs. Also, *THBS2*, a mediator of cell-cell and cell-matrix interactions, which suppresses angiogenesis and tumor growth, was downregulated in the co-culture conditions. Myofibroblast markers were expressed at higher levels in CAFs, whereas TGF-ß-related genes were highly expressed in all conditions (Figure 8*C*).

Together these data indicate that tumor organoids have an impact on the fibroblast phenotype by either enforcing CAF function or inducing pro-tumorigenic gene expression in NFs and, at the same time, suppressing tumor inhibitory signaling.

### Deconvolution of Bulk RNA Sequencing Data Reveals Distinct Gene Signatures in Organoids Grown in Different Conditions

To further validate the co-culture model, we employed a bioinformatics deconvolution strategy to our RNA-seq data by an unbiased approach using scRNA-seq analyses of primary CRC specimen from 2 published datasets.^12^^,^^46^ This strategy allowed for the calculation of the proportions of specific cell types present in the individual culture conditions.

**Figure 9:**
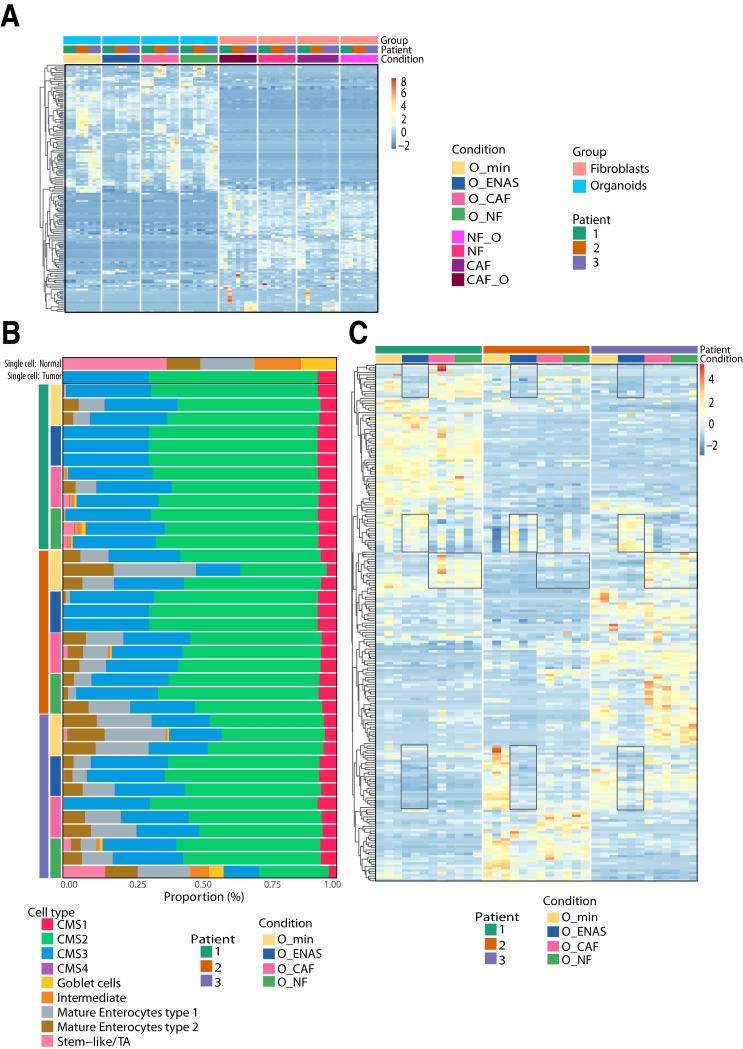
**Deconvolution of bulk RNA sequencing data based on scRNA-seq of primary CRC tumors.** (*A*) Hierarchical clustering of bulk RNA-seq gene expression levels of 137 marker genes from Lee et al^12^ as a reference used for the deconvolution of cellular proportions of organoids and fibroblasts cultivated in the indicated combinations and media. (*B*) Cellular proportions of organoids grown in minimal medium (O__min_), conventional organoid medium (O_ENAS), or together with NFs (O_NF) or CAFs (O_CAF), identified from deconvoluting bulk RNA-seq data using scRNA-seq of 23 patient samples (13 tumor, 10 normal) from Lee et al^12^ as a reference. We derived marker genes for normal epithelial cells including 5 sub-types (stem-like/TA, goblet cells, intermediate, mature enterocytes type 1, and mature enterocytes type 2) and from tumor cells reflecting the 4 CMS signatures (CMS1‒4). The top 2 rows in the graph display the cellular proportions of epithelial cells identified in primary normal tissues and tumors from Lee et al,^12^ respectively. Subsequent rows represent the individual epithelial and CMS proportions of the organoids grown in the respective conditions, sorted by patient (P1‒3). Different colors represent CMS1‒4 and epithelial cell sub-types. Three replicates are shown for each condition. Please note that the last row, showing a replicate of organoids grown in co-culture with NFs, has a very high proportion of epithelial-like cells, which might represent an outlier. (*C*) Heatmap of normalized gene expression values of marker genes used for the deconvolution of organoid cell proportions as described in (*B*). For each patient (P1‒3) and condition, 3 replicates are shown. Rectangles mark gene expression signatures of marker genes that are deregulated in organoids grown in ENAS compared to other conditions, or in co-cultures (O_NF, O_CAF) compared with mono-cultures.

First, we inferred marker genes from the Qian^46^ dataset to quantify cellular proportions of epithelial and fibroblast cells in the different culture conditions. Gene expression levels of all markers identified from the deconvolution displayed a clear separation of fibroblasts and epithelial organoid cells using hierarchical clustering, as expected (Figure 9*A* and Supplementary Table 7), confirming the purity of the cultures as already demonstrated with a small subset of specific marker genes above.

To define the cellular proportions of organoids grown in the 4 different conditions, we combined tumor-specific markers defining the CMS (CMS1‒4) and colon epithelial markers reflecting cellular subtypes and differentiation states from the Lee dataset^12^ (Supplementary Table 8). The selected markers allowed for the subclustering of different epithelial cell states, including stem-like transit amplifying (TA) and intermediate cells, goblet cells, and mature enterocytes from the original dataset (data not shown). As recently described, tumor epithelial cells are mainly represented by CMS2 and CMS3 gene expression profiles.^47^, ^48^, ^49^ Along these lines, we also observed enrichment of these subtypes in the organoid fractions (Figure 9*B*). Although we could observe interpatient heterogeneity among the organoid lines derived from the 3 individual patients, the ENAS condition mainly reflected the CMS2/3 tumor signatures. Upon cultivation in minimal medium (O__min_), a small proportion of differentiated mature enterocytes (type I and II) were apparent. Interestingly, co-culture with fibroblasts resulted in an increase of different cell type proportions, which was variable among the 3 patients but also included stem-like/TA and intermediate cells, goblet cells, and mature enterocytes. Hierarchical clustering of marker gene expression levels of organoids in the different conditions revealed patient-specific gene expression signatures; however, we also detected changes in marker gene expression based on the different conditions (Figure 9*C* and Supplementary Table 8). In 2 of the 3 patients, we identified a cluster of genes upregulated in both co-culture conditions including *MUC5AC*, *IFI6* and 27, *SERPINA1*, and *LYPD8*, which were previously reported to be involved in tumor progression and metastasis, tumor cell proliferation, and poor patient prognosis.^50^, ^51^, ^52^ In the ENAS condition, we found upregulation of *NMU*, *EREG*, and *SNHG12* genes. *NMU* and *EREG* encode for Neuromedin U and Epiregulin, respectively, both of which are involved in tumor invasion and progression.^53^^,^^54^ *SNHG12* encodes for a long non-coding RNA, which can regulate tumor cell invasion and metastases in endometrial cancer.^55^ On the other side, a cluster of genes related to differentiation were downregulated in the ENAS condition; these genes include *GUCA2A*, *GUCA2B*, and *KRT20*.

Thus, these data suggest that the co-cultivation of colon PDOs and fibroblasts induces a larger diversity of epithelial cell types as compared with the conventional system, where organoids are grown in monoculture and depend on stem cell niche factors.

### Identification of Functional Interactions Between Fibroblasts and Epithelial Cells

**Figure 10:**
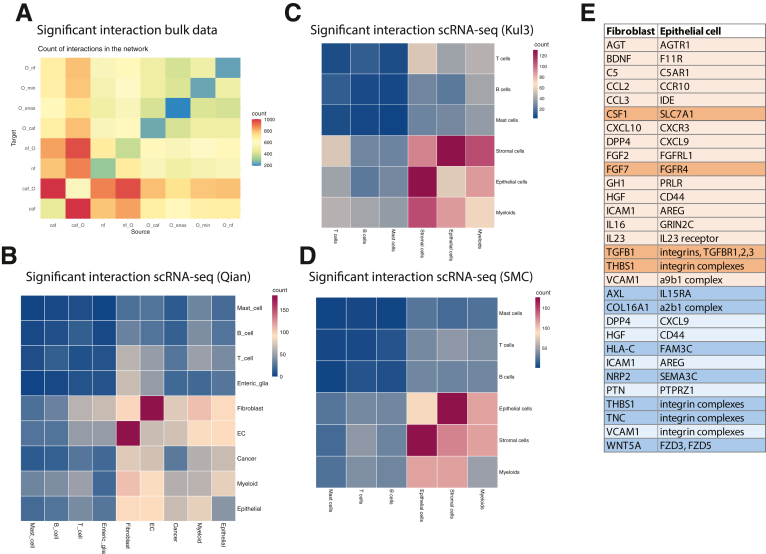
**Significant interactions of different cell types.** (*A*) Cell-cell interaction counts identified by CellphoneDB^56^ analysis of bulk RNA-seq data of purified fibroblast and organoid epithelial cells cultured in the indicated conditions (CAF, CAF mono-culture; CAF_O, CAFs co-cultured with organoids; NF, NFs mono-culture; NF_O, NFs co-cultured with organoids; O_CAF, organoids co-cultured with CAFs; O_ENAS, organoids cultured in ENAS medium, O_min, organoids cultured in minimal medium; O_NF, organoids co-cultured with NF). (B‒D) Cell-cell interaction counts of indicated cell types inferred from published datasets.^12^^,^^13^^,^^46^ (*E*) Significant interactions inferred from bulk RNA-seq data of CAF and organoid co-cultures that overlap with fibroblast secreted factors or proteins specifically upregulated in CAFs (*orange*, overlap with secretome proteins; *blue*, overlap with CAF proteome data; *darker shades* additional overlap with scRNA-seq fibroblast-epithelial cell interaction pairs).

To gain a deeper understanding of the relevant interactions between the different cell types in our co-cultures, we used the CellphoneDB tool^56^ to infer biologically relevant interactions. First, we took our bulk RNA-seq data, which were based on pure cell populations, to interrogate the crosstalk between fibroblasts (NF and CAF) and organoids (epithelial cells) (Figure 10*A*). We observed a high number of interactions among the different fibroblasts but also between fibroblasts and organoids, especially when fibroblasts were taken as the “source” and organoids as the “target” for the interaction pairs. Interestingly, when CAFs were co-cultured with organoids (CAF_O), the number of interactions was increased. Next, we assessed publicly available scRNA-seq datasets,^12^^,^^13^^,^^46^ which were derived directly from patient specimens, observing the most interactions of fibroblasts with stromal and endothelial cells (Figure 10*B–D*). When intersecting interactions of fibroblasts and epithelial cells from bulk data and scRNA-seq data, we identified 78 shared interaction pairs. These contained several fibroblast-specific collagen-integrin, fibronectin-integrin, or growth factor-receptor pairs, which are relevant for ECM remodeling and tumor promotion (data not shown). Interactions driven by the tumor cells were fewer and included ECM and signaling molecule interactions (data not shown). We then matched the significant interaction pairs driven by fibroblast factors, with factors identified in the secretome and proteome analyses described above (Figure 10*E*). Here, we found significant interactions of CAF-secreted cytokines and chemokines, such as CSF1, FGF7, or TGFB1, which were also confirmed in the scRNA-seq datasets. Proteome-identified targets included adhesion molecules and WNT5A. As a recurrent CAF-specific factor, we identified THBS1, which we further evaluated for its biological impact on CAF function in the organoid co-culture model.

### Thrombospondin 1 Expression is Increased in CAFs vs NFs and Affects the NF Phenotype

**Figure 11:**
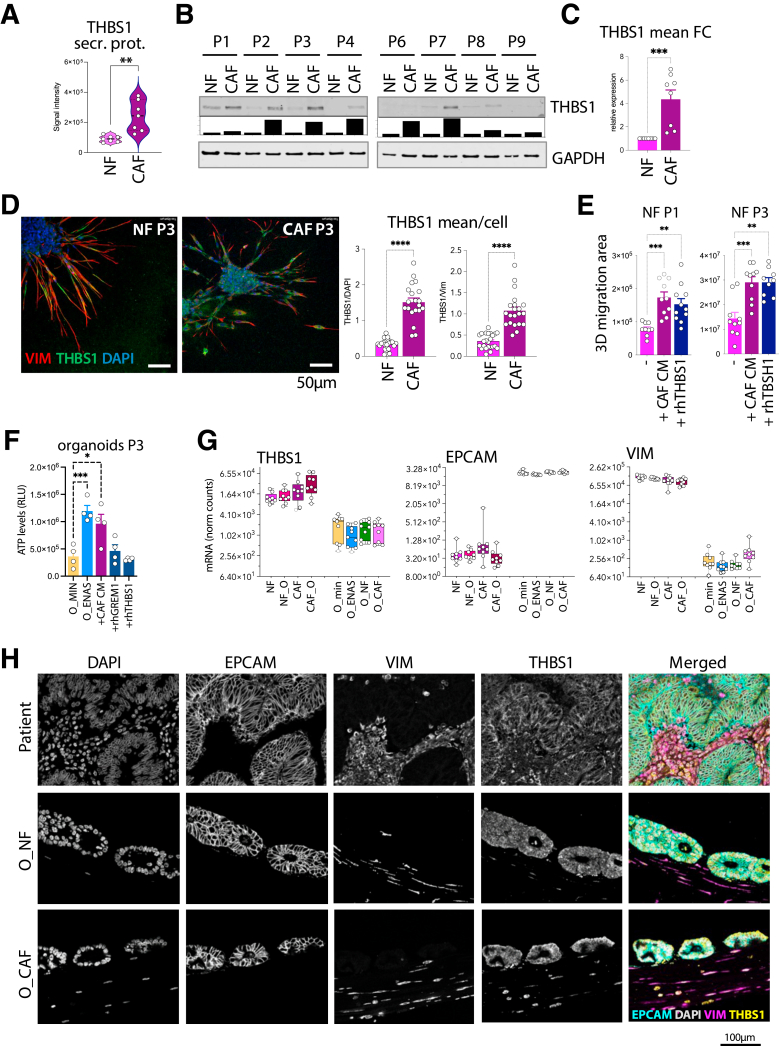
**THBS1 is elevated in CAFs compared with NFs and affects NFs phenotype.** (*A*) THBS1 levels in the secreted conditioned medium of NFs and CAFs; 7 pairs were analyzed by the Cytokine XL array. (*∗∗P* < .005; 2-tailed unpaired Student's *t* test). (*B*) Western blot analysis of THBS1 protein levels in CAFs compared with NFs in 8 patient pairs. Quantification is shown underneath normalized to GAPDH. *Bars* represent fold change CAFs to NFs. (*C*) Collective representation of WB analysis shown in (*B*) of the 8 patient NF/CAF pairs. (∗∗∗*P* < .001; 2-tailed unpaired Student's *t* test). (*D*) Immunofluorescence staining of THSB1 (*green*) in spheroids generated with NFs and CAFs. Vimentin (*red*) was used as a specific fibroblast marker, DAPI (*blue*) to stain for nuclei. (∗∗∗∗*P* < .0001; 2-tailed unpaired Student's *t* test). (*E*) Image analytical THBS1 quantification per cell is shown, normalized to DAPI or Vimentin expression. Each data point represents 1 cell. (∗*P* < .05, ∗∗*P* < .005, ∗∗∗*P* < .001; ordinary 1-way ANOVA.) (*F*) ATP levels as determined by CellTiter-Glo 3D viability assay after treatment of organoids for 7 days with hrTHBS1, human recombinant gremlin (hrGremlin), or CAF-CM. (∗*P* < .05, ∗∗∗*P* < .001; ordinary 1-way ANOVA.) (*G*) Transcript expression levels of THBS1, EPCAM, and Vimentin (VIM) in fibroblasts and organoids based on normalized counts from RNA-seq data. (*H*) Confocal microscopy images of tissue sections from patient formalin-fixed paraffin-embedded blocks or matched organoids/fibroblast cultures (O_NF or O_CAF). Antibodies against Vimentin (VIM, fibroblast marker), EPCAM (epithelial cell marker), or Thrombospondin 1 (THBS1) were used. DAPI was used to stain nuclei.

As we found THBS1 to be elevated in the secretome of CAFs compared with NFs (Figure 11*A*), we went on to confirm its expression at the protein level by Western blotting. Analysis of 9 patient-matched NF/CAF pairs confirmed that CAFs produce higher amounts of THBS1 (Figure 11*B–C*). The same finding was demonstrated by immunofluorescence staining of NF and CAF spheroids (Figure 11*D*). To evaluate the biological role of THBS1 for the fibroblast phenotype, we treated NF spheroids from 2 different patients with human recombinant THBS1 (hrTHBS1) protein and compared the motility phenotype to NF spheroids treated with CAF-CM or no treatment. Interestingly, both the CAF-CM and the hrTHBS1 treatments comparatively increased NF motility (Figure 11*E*). On the other hand, treatment of organoids with either hrTHBS1 or human recombinant Gremlin, another factor increased in CAFs, did not affect growth of organoids. Instead, their viability was enhanced by CAF-CM as compared with organoids grown in minimal medium (Figure 11*F*). Finally, expression of THBS1 was validated in primary tumors and compared with the levels produced in the co-cultures (Figure 11*G–H*). For this, we used sections from organoids with NFs or CAFs and sections from the original patient tumor. We stained the sections for THBS1, Vimentin (fibroblast marker), and EPCAM (epithelial cell marker). In agreement with the RNA levels for THBS1, the protein was detected in CAFs and NFs and also in the epithelial compartment in the primary tumors and in the co-cultures. Together, the data demonstrate consistent increase in THBS1 at the RNA and protein levels across patients and the reciprocal co-cultures, and its biological function for the CAF phenotype.

## Discussion

Organoid cancer models have shown great potential for precision medicine, and there are living biobanks available, which are rapidly growing.^37^^,^^57^^,^^58^ They show predictive power for patient responses to some of the standard-of-care therapies, such as irinotecan-based therapies in CRC.^4^ However, these models fail to predict patient response to oxaliplatin-based therapies in CRC for unknown reasons. One reason might be the lack of the tumor stroma in these models, which evidently is also strongly affected by chemotherapy^59^ and radiotherapy.^24^^,^^60^, ^61^, ^62^ Here, we present and thoroughly characterize a CRC PDO model integrating fibroblasts as the most abundant and important cell type in the TME. We provide substantial evidence that this advanced model is highly relevant to mimic the biological and physiological properties of in vivo tumor conditions.

First, the preservation of the CAF phenotype under in vitro culture is still a matter of debate, and thus is the use of these cultured cells in cancer models. By comparing early passage cultured NFs and CAFs from the same patients, we clearly demonstrate that bona fide CAF markers, which were identified in vivo, are preserved in culture. These include well-characterized tumor stroma genes such as *WNT2*,^40^ *WISP1*,^63^^,^^64^ *ITGB2*,^65^ *WNT5A*,*^45^* *VCAM1*,^39^^,^^41^ and *ICAM1*.*^66^* Moreover, by transcriptomic and proteomic approaches, as well as secretome analysis, we provide a list of differentially expressed molecules that are implicated in ECM organization as well as tumor and immune cell signaling. These molecular alterations were also apparent in the substantially elevated capacity of invasion/motility of CAFs into collagen gels. In line with our findings, CAFs have been described to display increased expression of matrix-modifying enzymes and show increased remodeling capacity,^67^^,^^68^ which is well-recapitulated in the expression data of the cultured NFs and CAFs patient pairs, presented here.

However, fibroblasts serve not only as remodelers of the matrix, but they also have multiple signaling functions in the crosstalk with the surrounding cells, supporting the development, spatial organization, and homeostasis of epithelial sheets in many organs.^69^ Indeed, we could recapitulate the supportive nature of stromal fibroblasts for organoid growth in vitro and demonstrate that both NFs and CAFs grown in collagen I gels are equally potent in facilitating the survival and expansion of organoid structures of epithelial tumor cells. Of note, this cell culture system relies on minimal organoid medium supplemented only with 1% FBS omitting all protein factors (eg, WNT3A, RSPO, Noggin, B27) and inhibitors (A-8301, SB212090) otherwise required for monoculture organoid expansion in basal membrane extract or Matrigel.^27^^,^^70^ Gene expression analyses revealed high levels of expression of WNT signaling molecules and growth factors, including EGF and FGF family members, as well as TGF-ß factors and Noggin in fibroblasts, suggesting that fibroblasts are capable of replacing standard media factors. The lack of inhibitors in the media is of special importance for an undisturbed establishment of the physiological crosstalk between the fibroblasts and the epithelial cells. For example, it is well-established that TGF-βR inhibition (by eg, A-8301 and Noggin) critically affects fibroblast biology,^71^^,^^72^ thus potentially masking physiologically relevant CAF phenotypes and crosstalk with the tumor cells. Moreover, the presence of myofibroblasts, which are generated by tumor cell-derived TGF-ß and are predominantly present at the invasive margin, were shown to promote HGF-dependent invasion of the cancer cells in squamous carcinoma cells.^73^ Co-culture systems with synchronous co-cultures from the start have been recently described in mouse and human and show similar results.^74^, ^75^, ^76^

Noteworthy, the independent establishment of the stromal and organoid cultures in this system provides one important intrinsic advantage, the possibility to analyze individual mono-cultures as well as the respective co-cultures, including the mutual crosstalk of tumor cells and fibroblasts. Among the induced genes in both NFs and CAFs upon co-culture, we found *IL6*, *ICAM1*, and *CXCL14*. IL6 is a cytokine that has been shown to induce EMT in gastric cancer cells.^77^ Intercellular adhesion molecule 1 (ICAM-1) has been implicated in several cancers including breast^78^ and metastatic melanoma^79^ by promoting cell invasion and transmigration, respectively. ICAM-1 was found to bind to MUC1 at the tumor leading edge, thus promoting cell invasion.^80^ CXCL14 was found elevated in colorectal,^81^ ovarian,^82^ prostate,^83^ and breast^84^^,^^85^ cancers. Stromal overexpression of CXCL14 in ovarian cancer was linked to poor patient survival via STAT3-dependent manner due to increased tumor cell proliferation.^82^ Taken all together, these data suggest that tumor cells are instructing CAFs on the production of specific molecules, which then, in turn, allow tumor cells to propagate and invade. The experimental setup here was designed to co-culture the cells for 4 days, which possibly is only the starting point where tumor cells transform fibroblasts into a supportive tumor entity, and longer cultures would possibly induce larger changes in the fibroblasts. On the other hand, the co-culturing conditions with either NFs or CAFs significantly upregulated genes in organoids related to ECM organization and collagen formation and biosynthesis, which suggests that phenotypic alterations are not limited only to fibroblasts.

Strong support for the physiological meaningfulness of the model presented here comes from histomorphological analyses of the co-cultures. The level of similarity of marker protein expression such as CEACAM1, TFF3, OLFM4, and MUC1 and presence of mucus was higher between the patient tissue and the co-cultures as compared with organoids alone – either in minimal or ENAS medium. Furthermore, mucus presence in tumors is usually related to worse patient outcome,^86^ and thus, its presence might be relevant in cultures, when drug response is tested. Also, RNA-seq results when juxtaposed to single-cell sequencing data from CRC supported the presence of various cell types in the fibroblast co-cultures as observed in ex vivo tumors.^12^^,^^13^ This demonstrates that organoid expansion medium is indeed enriching stem cells as expected,^27^ whereas the heterogeneity of cell types is inferior to that in organoids grown with fibroblasts exemplified by, for example, the presence of goblet cell markers or tuft cell markers in this group. Organoids in minimal medium showed an increase in differentiation markers such as *KRT20*, *CLDN7*, and *VIL*^87^, ^88^, ^89^ as anticipated but failed to show diversity. Thus, the culture system presented here shows high levels of tumor cell heterogeneity, which is important for cancer progression and drug response and could hence be of important translational impact.^90^, ^91^, ^92^

Deconvolution of the bulk RNA-seq data of this study demonstrated expression of genes found in primary tumors. Particularly, the genes *MUC5AC*, *IFI6* and *IFI27*, *SERPINA1*, *LYPD8*, and *CHP2* were predominantly expressed in organoids co-cultured with fibroblasts but not in monocultures. As these genes are essential to promote tumor growth and metastases formation, these data agree with previous reports that fibroblasts enhance tumor progression.^93^, ^94^, ^95^ Furthermore, this demonstrates that there is considerable difference in expression patterns in PDOs when they are grown as a mono- vs co-culture with fibroblasts. These differences need to be considered when performing drug screens with specific inhibitors or searching for altered signaling pathways in an in vitro system.

The cellular crosstalk between fibroblasts and epithelial tumor cells was defined based on CellPhoneDB^56^ analyses, highlighting the importance of ECM components, cell adhesion molecules, cytokines, and growth factors. Interestingly, we identified THBS1, which fulfills a multitude of functions in the tumor microenvironment, including immunity and chemotherapy resistance,^96^^,^^97^ as a potent factor regulating the motility phenotype of CAFs. Recent literature described THBS1 as a TGF-ß target and a factor regulating tumor cell invasion in glioblastoma and oral squamous carcinoma.^98^^,^^99^

Taken together, we established a system that closely recapitulates the primary tumor biology in its morphology and gene expression patterns and allows studying relevant tumor-stroma communication. Future work would be needed to dissect expression changes within single cells in both the tumor and the stromal compartment, ideally involving the full repertoire of cells including the fibroblasts, immune cells, and possibly endothelial cells. Additionally, it will be important to validate this culture system for its accuracy to recapitulate patients’ responses to standard-of-care therapy. This could provide more precise treatment options and allow for the stratification of patients to optimized therapy solutions. In addition, we foresee a high potential of this system to screen for new therapeutic options that target both tumor cells and the surrounding stroma, which might help to limit therapy resistance and result in improved treatment outcome.

## Methods

### Study Design

The aim of this study was to generate a PDO model from CRC tumor specimens including stromal fibroblast cells isolated from the same patient. For this purpose, we used primary patient tumors or adjacent normal mucosa to establish and characterize organoids and fibroblasts – both cancer-associated or normal. In total, we developed NF-CAF pairs from 12 individual patients and a complete set of organoids and matched NF-CAFs from 6 of 12 patients. The in-depth molecular characterization of the model was done in comparison to classical organoid mono-cultures and a control condition, using 3 individual patient samples, representing 3 biological replicates. All experiments were further done in at least 3 independent replicates.

### Ethics Approval for the Patient Material

Patient material was obtained upon signed consent, and the experiments were performed according to the “Good Scientific Practice Guidelines” of the Medical University of Vienna, as well as the latest “Declaration of Helsinki.” The ethical protocol has been reviewed and approved by the ethics review board of the Medical University of Vienna (Votum N# 1248/2015).

### Statistics

Statistical analyses were performed with Prism software (GraphPad, Version 9). For the study, either the 2-tailed Student's *t* test or 1-way analysis of variance (ANOVA) were used, as indicated. A *P* value < .05 was set as a threshold of significance.

### Organoid and Fibroblast Isolation

Tumor pieces were processed either fresh or viably frozen. Every piece was washed 3 times with phosphate buffered saline (PBS) and then minced into 1-mm pieces. Pieces were transferred into a 15-mL tube and incubated in 5 mL DMEM 10 % FBS and 1 mg/mL collagenase type I at 37 ° for 1 hour. Then the digest was applied onto a 70-μm strainer. The single-cell suspension was washed 3 times with PBS, and in the last wash, the pellet was divided into 2 – one-half was embedded into Matrigel on a 24-well plate and the other one-half was plated on a 10-cm dish with EGM2-MV (PromoCell Cat# C22022) medium for fibroblasts growth.

### Organoid and Fibroblast Culture

Organoids were cultured in 30-μL Matrigel droplets in 24-well plates and passaged every 3 to 4 days. They were grown in organoid medium as previously described.^27^ To enrich for tumor cells, Wnt3a and Rspondin 1 were omitted from the medium. After isolation, fibroblasts were grown in EGM2-MV (PromoCell Cat# C22022) medium and passaged every 3 to 4 days in T75 flasks. Organoids were used up to passage 20 (P20) and fibroblasts in passages 3 to 6 (P3‒6).

### Mutational Profile of Organoids

The mutational profile of the organoids and patients was evaluated using the Ion AmpliSeq Cancer Hot Spot Panel v2 (Thermo Fisher, Cat# 4475346), which includes the following genes: *ABL1, EGFR, GNAS, KRAS, PTPN11, AKT1, ERBB2, GNAQ, MET, RB1, ALK, ERBB4, HNF1A, MLH1, RET, APC, EZH2, HRAS, MPL, SMAD4, ATM, FBXW7, IDH1, NOTCH1, SMARCB1, BRAF, FGFR1, JAK2, NPM1, SMO, CDH1, FGFR2, JAK3, NRAS, SRC, CDKN2A, FGFR3, IDH2, PDGFRA, STK11, CSF1R, FLT3, KDR, PIK3CA, TP53, CTNNB1, GNA11, KIT, PTEN, VHL.*

### Viability Assay

For assessing the viability of organoids and fibroblasts, the cells were plated in 10-μL Matrigel drops on a 96-well plate. Each drop contained either fibroblasts alone (1 × 10^4^ cells per well) or organoids (2 × 10^3^ cells per well) or both. The numbers of organoids and fibroblasts were selected after optimization procedures using 1000, 2000, or 3000 organoid cells and 5000, 10,000, or 20,000 fibroblast cells. To each well, 90 μL of medium was added, and medium was exchanged every other day. After 7 days of culturing, 100 μL of CellTiter-Glo 3D Cell Viability Assay (Promega Cat# G9681) was added to each well and incubated for 5 minutes on a shaker. Luminescence was measured using Synergy HTX Multi-Mode Microplate Reader.

### Organoid and Fibroblast Treatments

For treatment of the organoids with selected inhibitors, they were plated as for viability assays described above. Treatments were performed with fibroblast conditioned medium, collected in advanced DMEM/F12 containing 1% FBS from patient-matched CAFs for 48 hours and prior mixed 1:1 with fresh medium before addition to the organoids. For inhibition of the EGF pathway, EGF inhibitors allitinib (1 μM) or erlotinib (1μM) were added to the medium. The inhibitors A8301 and SB202190 were used at 500 nM and 3μM, respectively. Cultures were treated for 7 days, and the medium was changed every other day. On the last day, viability was measured using the CellTiter-Glo 3D assay.

For treatments with hrTHBS1, fibroblast spheroids from normal fibroblasts were pretreated for 24 hours, either with at a concentration of 1 μg/mL THBS1 or with conditioned medium from CAFs, which was collected as described above.

### Immunohistochemistry Staining and Quantification

For immunohistochemical stainings, co-cultures were prepared. Fibroblasts were embedded in collagen gels as described (300,000 fibroblasts per 300 μL gel). When gels had solidified, they were coated with 10 μL Matrigel for 20 minutes and lifted on cell culture inserts, where the Matrigel coating was upwards. On top, we plated organoids at 50 organoids per gel. Numbers for organoids and fibroblasts were chosen based on optimization procedures described above. To each well, 1.5 mL medium was added underneath to ensure the organoids were grown at the air-liquid interphase. The co-cultures were grown for 1 week and then fixed in 4% paraformaldehyde for 20 minutes. Then they were embedded in paraffin and sectioned at 2-μm sections for staining. Antibodies used were anti- CDX2 (Biogenex Cat#NC1701134), anti-Ki67 (Ventana Medical Cat#790-4286), anti-CK20 (Ventana Medical Cat# 790-4431), anti-Fibronectin (Dako Diagnostics Cat#A0245), anti-CEACAM1 (Cell Signaling, Cat#5441), anti-TFF3 (Sigma Aldrich, Cat# HPA 035464), anti-CD44 (Cell Signaling, Cat#37259), anti-OLMF4 (Sigma Aldrich, Cat# HPA 077718), and anti MUC1 (Ventana, Cat# 790-4574). Staining intensity was quantified using Fiji software as described earlier.^100^

### Organoid Outgrowth

To measure organoid growth over time depending on fibroblasts number, cultures were prepared as for immunohistochemical staining, and images of the cultures were taken for 7 consecutive days. The growth area of the individual organoids was measured in ImageJ and plotted in Graphpad Prism 9.

### Immunofluorescence Staining

Collagen gels and tissue sections were washed in PBST (0.5% Tween 20) 3 times for 10 minutes each and then incubated with 2% BSA (in PBST) for 3 hours at room temperature. After blocking, the following primary antibodies were added for incubation over night at 4 °C anti-THBS1 (Santa Cruz, sc-376994), anti-Vimentin (Abcam, ab194719), and anti-EPCAM (Santa Cruz, sc-59783). After primary antibody incubation, samples were washed with PBS 3 times, and secondary antibodies (anti-mouse, Invitrogen A-11032; anti-rabbit, Invitrogen, A-11034) were added for 1 hour at room temperature. Samples were washed 3 times with PBS and incubated with DAPI for 20 minutes at room temperature.

### 3D Migration Assay

NFs and CAFs were trypsinized and counted. For formation of spheroids, 500 cells were plated per well in U-bottom 96-well plates in 100 μL DMEM 5% FBS/methylcellulose 5%. Plates were incubated for 5 hours in the incubator to allow spheroids to form. Then spheroids were collected, washed with PBS, and embedded into collagen gels from rattail collagen I at 2 mg/mL. When the gels were solidified, they were moved to 24-well plates, and spheroids were grown for 24 hours and imaged thereafter. Quantification was performed using Fiji software by drawing the outgrowth area of each spheroid and subtracting the middle spheroid area; single cells and number of protrusions were counted separately. The data were plotted in GraphPad Prism.

### Cytokine Arrays

For evaluating the secretome of NFs and CAFs, 2 × 10^6^ cells were plated per T75 flask in EGM2-MV medium. The next day, the medium was exchanged with DMEM/1% FBS and incubated for 48 hours. Then, medium was collected and centrifuged for removal of floating cells. From that, 1 mL was used for further analysis. The assay was performed according to the manufacturer’s instructions (R&D systems Cat# ARY022B). Readout of fluorescence was performed on Odyssey DLx, and the fluorescence intensity was quantified with Image Studio Lite Ver 5.2 software.

### RNA Collection and Sequencing

For RNA collection, organoids and fibroblasts were grown as co-cultures onto inserts with a 0.45-μm membrane. Fibroblasts were embedded into collagen gels from rat tail collagen I at a concentration of 2 mg/mL. The gels were casted onto one side of the membrane, and organoids were plated in 100 μL Matrigel drops on the other side of the membrane. The co-culture was grown in 6-well plates, where the fibroblast gels were positioned on the lower side and the organoids on the top. To each culture, 1.5 mL medium was added, either ENAS medium or 1% FBS in Advanced F12/DMEM, and the cells were cultured for 4 days. After 4 days, the gels were lyzed, and total RNA was collected using QIAGEN RNeasy kit (Cat# 74106). RNA quantity was evaluated with a Qubit Fluorometer, and 200 ng RNA were used for bulk RNA-seq.

Sequencing libraries were prepared using the NEBNext Ultra II Directional Kit with polyA selection module (New England Biolabs). In brief, total RNA was used as an input for the mRNA selection with oligo-dT paramagnetic beads. mRNA was then fragmented and converted into double stranded cDNA. Following universal adapter ligation, samples were polymerase chain reaction-barcoded using dual indexing primers. Samples were sequenced in depth of 30 to 52 million paired-end 2 × 150 bp reads on Illumina NovaSeq 6000 sequencer (Illumina).

### Bioinformatic Analysis of RNA-seq Data

Bcl files were converted to Fastq format using bcl2fastq v. 2.20.0.422 Illumina software for base calling. Quality check of raw paired-end fastq reads was carried out by FastQC (http://www.bioinformatics.babraham.ac.uk/projects/fastqc/). The adapters and quality trimming of raw fastq reads was performed using Trimmomatic v0.36^101^ with settings CROP:250 LEADING:3 TRAILING:3 SLIDINGWINDOW:4:5 MINLEN:35. Trimmed RNA-Seq reads were mapped against the human genome (hg38) and Ensembl GRCh38 v.94 annotation using STAR v2.7.3a^102^ as splice-aware short read aligner and default parameters except --outFilterMismatchNoverLmax 0.1 and --twopassMode Basic. Quality control after alignment concerning the number and percentage of uniquely and multi-mapped reads, rRNA contamination, mapped regions, read coverage distribution, strand specificity, gene biotypes, and polymerase chain reaction duplication was performed using several tools namely RSeQC v2.6.2.^103^ Picard toolkit v2.18.27 (http://broadinstitute.github.io/picard), and Qualimap v.2.2.2^104^ and BioBloom tools v 2.3.4-6-g433f.^105^ The differential gene expression analysis was calculated based on the gene counts produced using RSEM tool v1.3.1^106^ and further analyzed by Bioconductor package DESeq2 v1.20.0.^107^ Data generated by DESeq2 with independent filtering were selected for the differential gene expression analysis due to its conservative features and to avoid potential false-positive results. Genes were considered as differentially expressed based on a cut-off of adjusted *P*-value ≤ .05 and log2(fold-change) ≥1 or ≤−1. Clustered heatmaps either for organoids or fibroblasts sample fractions were generated from selected top differentially regulated genes using the R package pheatmap v1.0.10.^108^ The gene selection was performed separately for fibroblasts and organoids, where differential expression analysis was carried out for all possible condition pairs followed by union of significantly differentially expressed genes from all comparisons. All heatmaps were based on logarithmic DESeq2 normalized gene expressions with removed patient batch effect and further normalized on Z-score. We utilized hierarchical clustering to sort genes into individual clusters. Volcano plots were produced using ggplot v3.3.3 package (https://doi.org/10.1002/wics.147), and MA plots were generated using ggpubr v0.4.0 package version 0.1 7 (2018) (https://CRAN.R-project.org/package=dply.). PCA plots were based on DESeq2 normalized gene expression, further adjusted by variance-stabilizing transformation, as well as by removing patient batch effect and utilizing prcomp function from stats v3.6.3 package (https://www.R-project.org/). Reactome pathway analyses including pathways significance bar and dot plots as well as network connection figures were executed by adapting of clusterProfiler v3.12.0 package.^109^

### Proteomics

Proteins were precipitated with 4 volumes of ice-cold acetone at −20 °C overnight. After centrifugation at 10000 × g for 30 minutes, the supernatant was discarded, and the remaining pellets were washed with 150 μL 80% ice-cold acetone. After centrifugation at 10000 × g for 5 minutes, the supernatant was discarded, and the remaining acetone was allowed to evaporate. The protein pellet was resuspended in 50 μL 8 M urea in 50 mM ammonium bicarbonate, and the protein concentration was determined using a Bradford assay (BioRad). Proteins were reduced using 10 mM dithiothreitol for 30 minutes at room temperature, alkylated with 20 mM iodoacetamide for 30 minutes at room temperature in the dark, and the remaining iodoacetamide was quenched with the addition of 5 mM dithiothreitol. Aliquots of 60 μg protein were diluted to 4 M urea using 50 mM ammoniumbicarbonate, then 600 ng Lys-C were added, and the samples were incubated at 25 °C for 2 hours. After dilution to 1 M urea 600 ng trypsin were added, and the samples were incubated at 37 °C overnight. The samples were acidified with 0.5% trifluoroacetic acid, and one-fifth of each sample was desalted using C18 Stagetips.^110^ Sample quality and concentration was determined by injecting 3% of each sample on a HPLC-UV system at 214 nm, using a monolithic C18 column for peptide separation.

An estimated 600 ng peptides were separated on an Ultimate 3000 RSLC nano-flow chromatography system (Thermo-Fisher), using a pre-column for sample loading (Acclaim PepMap C18, 2 cm × 0.1 mm, 5 μm, Thermo-Fisher), and a C18 analytical column (Acclaim PepMap C18, 50 cm × 0.75 mm, 2 μm, Thermo-Fisher), applying a segmented linear gradient from 2% to 35% and finally 80% solvent B (80 % acetonitrile, 0.1 % formic acid; solvent A 0.1 % formic acid) at a flow rate of 230 nL/min over 120 minutes. Eluting peptides were analyzed on a Q Exactive HF-X Orbitrap mass spectrometer (Thermo Fisher), which was coupled to the column with a nano-spray ion-source using coated emitter tips (PepSep, MSWil).

The mass spectrometer was operated in data-dependent acquisition mode; survey scans were obtained in a mass range of 375-1500 m/z with lock mass activated, at a resolution of 120k at 200 m/z and an AGC target value of 3E6. The 25 most intense ions were selected with an isolation width of 1.2 m/z, fragmented in the HCD cell at 28% collision energy, and the spectra recorded for maximum 54 ms at a target value of 1E5 and a resolution of 15k. Peptides with a charge of +2 to +6 were included for fragmentation, the peptide match and the exclude isotopes features were enabled, and selected precursors were dynamically excluded from repeated sampling for 30 seconds.

Raw data were processed using the MaxQuant software package version 1.6.0.16 as described^111^ and the Uniprot human reference proteome (www.uniprot.org, release 2018_08), as well as a database of most common contaminants. The search was performed with full trypsin specificity and a maximum of 2 missed cleavages at a protein and peptide spectrum match false-discovery rate of 1%. Carbamidomethylation of cysteine residues were set as fixed; oxidation of methionine and N-terminal acetylation were set as variable modifications. Label-free quantification the “match between runs” feature and the LFQ function were activated ‒ all other parameters were left at default.

MaxQuant outputs were filtered for potential contaminants and reverse hits, and in case they were only identified by site. Additionally, only protein groups that were quantified in at least 75% of the samples in at least one condition (ie, 3 of 4 in CAF or NF) have been included for further downstream differential protein expression analysis using custom R-scripts, mainly using functions from the DEP package.^112^ After intensity normalization by variance stabilizing transformation^113^ and confirming left-censored nature of missing values, remaining missing values were imputed applying the MinProb method.^114^ Statistical testing for differentially expressed proteins was done by using functions implemented by the DEP package, making use of protein-wise linear models combined with empirical Bayes statistics.^112^ Secretome measurements were analyzed for differential intensity similarly; missing values were not present.

Subsequent pathway enrichment analyses were performed via active subnetworks using the pathfindR package.^115^ As background protein-protein interaction network, we selected STRING,^116^ as input only significantly differentially expressed proteins with a *P*-value lower than .05 and an absolute log2-fold-change higher than 1 (ie, 2-fold) and differentially secreted proteins with a *P*-value lower than .05 for secretome data were used.

### Western Blot Analysis

Fibroblasts were plated and cultured on 10-cm dishes with 1 × 10^6^ cells per dish for 48 hours. The cells were washed with ice-cold PBS and lyzed with radioimmunoprecipitation assay buffer. After scraping, the lysate was centrifuged at 4 °C for 10 minutes at maximum speed, and the supernatant was used to measure protein concentration with Bradford assay. A total of 40 μg were loaded onto SDS gels for electrophoresis. Proteins were transferred onto nitrocellulose membrane using a BioRad Turbo blot apparatus, and after blocking, primary antibodies against MYLK (Santa Cruz, sc-365352), THBS1 (Santa Cruz, sc-376994), or VCAM1 (Santa Cruz, sc-18864) were prepared and added onto the membranes overnight. Secondary fluorescently labeled antibodies were added for 1 hour, and membranes were imaged using a Licor device.

### Deconvolution

We use a modified version of the SCDC library^117^ to calculate the proportions of the bulk RNA-seq with different scRNA-seq as a reference. The general procedure allows the deconvolution of bulk RNA-seq expression samples based on a scRNA-seq dataset with a multiple reference cell-type clustering, avoiding pre-defined marker genes. These are the steps in the procedure: first, a signature matrix is built based on the cell type scRNA-seq reference. Next, the cell-type proportions for the bulk samples are recovered using a tree-guided procedure and a weighted non-negative least square regression framework over the marker genes, weighting each gene by cross-subject and cross cell variation. The method defines an observed bulk gene expression $Y\in R^{NxM}$ for N genes across M samples, each containing K cell types. The deconvolution constructs 2 non-negative matrices $B\in R^{NxK}$ (signature matrix) and $P\in R^{KxM}$ (joint proportions of the K cell type by sample) such that:

Y≈BP

The SDCC library has been modified to allow parallelism, matrix sparsity, and selection of marker genes using a dynamic threshold for each cluster in the single-cell reference matrix. The native approach for the selection of marker genes was completed with a global threshold using a Wilcoxon test between each cell type and the remaining dataset. This creates an unbalanced set of marker genes, where cell types have either a very high number, few, or zero markers. To overcome this limitation, this has been substituted by a process with multiple bootstrapping samples on all clusters, selecting n_b_ clusters as background and then performing a Wilcoxon test over each sampling and cluster of interest. Last, using DBSCAN to perform outlier analysis, the procedure selects an optimum number of genes that behave as markers for the specific cell type.

### Deconvolution of Samples

We used 73 bulk data samples divided into 2 groups: 36 organoids and 37 fibroblasts. As a reference, we used the following 2 scRNA-seq datasets: SMC dataset:^12^ This contains data from scRNA-seq of unsorted CRC single cells of 23 patients, of which 13 are from tumor samples and 10 are from normal samples. The authors originally identified 5 cell types (stromal, Myeloid, Mast, T, B, and Epithelial cells) through clustering, and 4 consensus molecular subtypes (CMS1, CMS2, CMS3, and CMS4). After filtering just the epithelial cells, we recovered 18,539 cells. Of these, 1070 corresponded to normal epithelial cells with 5 sub-cell types (stem-like/TA, goblet cells, intermediate, mature enterocytes type 1, and mature enterocytes type 2) and 17,469 tumor cells with the 4 CMS clusters described earlier. To run the deconvolution with the tree-guided procedure, we divided the 9 cell types into the following groups: Cluster group 1: CMS1, CMS2, CMS3 and CMS4; Cluster group 2: stem-like/TA, goblet cells, intermediate; and Cluster group 3: enterocytes type 1, mature enterocytes type 2. Using an initial pseudo bulk data simulated based on the single-cell subset, we determined the optimal parameters for deconvolution selecting n_b_ = 4 background cell clusters, selected n_bs_ = 100 bootstrapping samples, and limited the number of required marker genes per cluster to be between N_min_ = 28, N_max_= 35. With the optimal parameters, we obtain a Pearson correlation value of >0.999 between the true proportions and the estimated proportions and a sum of residuals < 3.6e−28. After the simulation, we ran the deconvolution with the actual bulk data, and we obtained 220 marker genes and a deviance value of 8.97 (0.12 on average for each of the 73 samples). Pan-cancer blueprint single-cell profiling dataset:^46^ This dataset includes data about CRC from 7 patients, 14 normal tissues, and 7 with cancer. The study includes 9 cell types clustered (epithelial, fibroblast, B cell, cancer, endothelial, enteric glia, mast cells, myeloid, and T cell). After filtering, we retrieved 4 cell types: epithelial, fibroblast, cancer, and endothelial defined by 44,684 cells, where 14,058 were normal epithelial cells with 5 cell types and 30,626 tumor cells. To run the deconvolution with the tree-guided procedure, we grouped the 4 cell types as follows: Cluster group 1: epithelial, fibroblast; and Cluster group 2: cancer, endothelial. Using a pseudo bulk data simulated based on the single-cell subset, we determined the optimal parameters for deconvolution selecting n_b_ = 2 background cell clusters, chosen n_bs_ = 100 bootstrapping samples and setting the number of required markers genes per cluster to be between N_min_ = 28, N_max_= 35. With the optimal parameters, we obtain a Pearson correlation value of >0.9059 between the estimated proportions and the true proportions and a sum of residuals <340.90 (170,45 in average for normal and tumor samples). After the simulation, we executed the deconvolution with the actual bulk data, and we obtained 137 marker genes and deviance of 7774.67 (106.50 on average for each of the 73 samples). Finally, we used the marker genes to visualize the normalized expression values by sample.

### Cell-cell Interactions With CellPhoneDB

For the identification of significant cell-cell communication mediated by ligand-receptor complexes between fibroblasts and epithelial cancer cells, we used the CellPhoneDB library V3,^56^ which is a public repository of ligands, receptors, and their interactions with manual curation of specific families of proteins involved in cell-cell communication. We used 3 different single-cell datasets (KUL3, SMC, and Qian) described before^12^^,^^46^ to execute the main 2 functions: statistical_analysis and degs_analysis with the parameter iterations equal to 1000. Also, we generated and analyzed the different default files and visualizations that are suggested as output in the protocol to find relevant interactions between cell types.

Finally, we used the function statistical_analysis with our bulk expression data, since the authors implemented the capability to analyze bulk data following the same idea.
